# The role of the FliD C-terminal domain in pentamer formation and interaction with FliT

**DOI:** 10.1038/s41598-017-02664-6

**Published:** 2017-06-30

**Authors:** Hee Jung Kim, Woongjae Yoo, Kyeong Sik Jin, Sangryeol Ryu, Hyung Ho Lee

**Affiliations:** 1 Department of Chemistry, College of Natural Sciences, Seoul National University, Seoul, 08826 Korea; 2 Department of Bio & Nano Chemistry, Kookmin University, Seoul, 136-702 Korea; 3 Department of Food and Animal Biotechnology, Department of Agricultural Biotechnology, and Research Institute for Agriculture and Life Sciences, Seoul National University, Seoul, 08826 Korea; 4 Pohang Accelerator Laboratory, Pohang University of Science and Technology, 80 Jigokro-127-beongil, Nam-Gu, Pohang, Kyungbuk 37673 Korea; 5 Center for Food and Bioconvergence, Seoul National University, Seoul, 08826 Korea

## Abstract

Flagellar biogenesis is controlled by a negative feedback loop. When FliD was secreted at the late step of flagellar assembly, the FliD-FliT complex disassembled and free FliT bound to the FlhDC complex, a master regulator of flagellar biogenesis, subsequently inhibiting the overall expression of flagellar proteins. In this study, we analyzed the role of the FliD C-terminal domain in pentamer formation and interaction with FliT. Our study showed that the FliD L443R mutant exists as a monomer in solution, indicating that the Leu443 residue of FliD, which contributes to its interaction with FliT, plays a crucial role in the pentameric oligomerization of FliD. Consistently, the increased levels of free FliT proteins caused by FliD L443R mutation had negative effects on the gene expression of flagellar synthesis and reduced the expression of flagellar proteins. The lengths of flagella in each cell were significantly reduced in L443R mutant strain, suggesting that normal flagellar biogenesis was impeded. These results suggest that the C-terminal domain of FliD plays a crucial role in the pentameric oligmerization of FliD and the binding of FliT to the C-terminal domain of FliD is critical to inhibit the premature assembly of the FliD pentamer in the cytosol.

## Introduction

The flagellum is an essential organelle that is necessary for bacterial motility and is composed of three parts: a basal body embedded in the cell wall (a rotary motor) and two external structures, a short proximal hook (a universal joint), and a long helical filament (a propeller, polymerized from the flagellin proteins)^1^. During the stages of the flagellar assembly process, the flagellar genes are expressed in response to morphological development of flagellar assembly^2^. There are three promoter classes in transcriptional hierarchy and several regulation mechanisms have been identified^3^. The FlhD_4_C_2_ complex, a master regulator of flagellar biogenesis, is at the top of the flagellar transcriptional hierarchy and plays a role in activating the synthesis of class 2 flagellar genes including those coding for the hook–basal body subunits and regulatory proteins^4^. In addition to FlhD_4_C_2_ complex, FliZ and FliT also act as positive and negative regulatory factors, respectively, for class 2 flagellar gene expression^5^.

Flagellum is assembled in a series of sequential steps beginning with the basal body formation and completing with the filament elongation^1^. The σ^28^-FlgM regulatory circuit plays a key role in controlling class 3 flagellar gene expression^6^. This regulatory circuit enables class 3 genes to be expressed only when the hook-basal body (HBB), a key intermediate during flagellum assembly, is complete^6^. Prior to the complete construction of HBB, the proteins required for forming it are secreted. However, once HBB is complete, FlgM, an anti-σ^28^ factor, is secreted from the cell through HBB, allowing σ^28^ factor to activate the expression of class 3 flagellar genes responsible for synthesizing the long helical filament^6, 7^. This regulatory hierarchy is well-conserved among Gram-negative enteric bacteria with peritrichous flagella^8^.

Following the HBB completion, HAP2 (hook-associated protein 2, also called FliD) forms a cap on the top of the hook-HAP1-HAP3 complex prior to the filament elongation^9^. Then, flagellin subunits passing through the channel polymerize below the cap one after another to form the long helical filament^10^. Because of the small diameter of the export channel (25–30 Å), it was thought that the HAPs such as FlgK (HAP1), FlgL (HAP3), and FliD (HAP2) are exported as partially unfolded monomers^11, 12^. To transport unfolded monomers of HAPs though the HBB, flagella-specific chaperones such as FlgN and FliT are required^13^. FlgN functions as an export chaperone for FlgK and FlgL, while FliT binds to the flagellar filament capping protein FliD^14^. In *Salmonella* Typhimurium, FlgN and FliT form homodimers, and prevent oligomerization of HAPs by direct interaction using their C-terminus or N-terminus, respectively^14^, indicating that FlgN and FliT are substrate-specific flagellar chaperones that prevent oligomerization of the HAPs by binding to their helical domains before export from the cell^13^.

During the final stage of flagellar assembly, one might expect that the expression of flagellar proteins is repressed to prevent the cell from using energy to synthesize unnecessary proteins. The overall production of flagellar proteins is reduced by sensing flagellar morphology via FliD-FliT complex. Formation of the FliD-FliT complex was known to play diverse roles; first, by inhibiting the premature assembly of the FliD pentamer in the cytosol^13^; second, by facilitating the secretion of FliD from cells by binding to the FlhA cytoplasmic domain only in complex with FliT^15^; third, by increasing flagellar biosynthesis by enhancing FlhD_4_C_2_ activity^16^. FlhD_4_C_2_ activity is negatively regulated by FliT, thus, FliD was proposed to act as an anti-anti-regulator, in a manner similar to the σ^28^-FlgM regulatory circuit originally by Yamamoto and Kutsukake^17^. Indeed, FliD acts as an anti-anti-FlhD_4_C_2_ factor because the FliD and FlhD_4_C_2_ binding sites on FliT overlap^18^. FliT also interacts with the FlhC in FlhD_4_C_2_ complex and increases the presentation of the FlhC recognition region to ClpX, resulting in FlhC proteolysis^17, 19^.

Recently, solution structure of the FliD-FliT complex, containing a smaller fragment of FliD compared to our crystal structure, was reported^16^. However, it remains unclear why the binding of FliT to the C-terminal domain of FliD is critical to inhibit the premature assembly of the FliD pentamer in the cytosol. To further analyze the detailed interactions at the FliD-FliT interface and understand the negative feedback loop in molecular details, we performed structural and functional analysis of the FliD-FliT interaction by multi-experiment analysis.

## Results and Discussion

### Interaction between StFliD and StFliT

**Figure 1:**
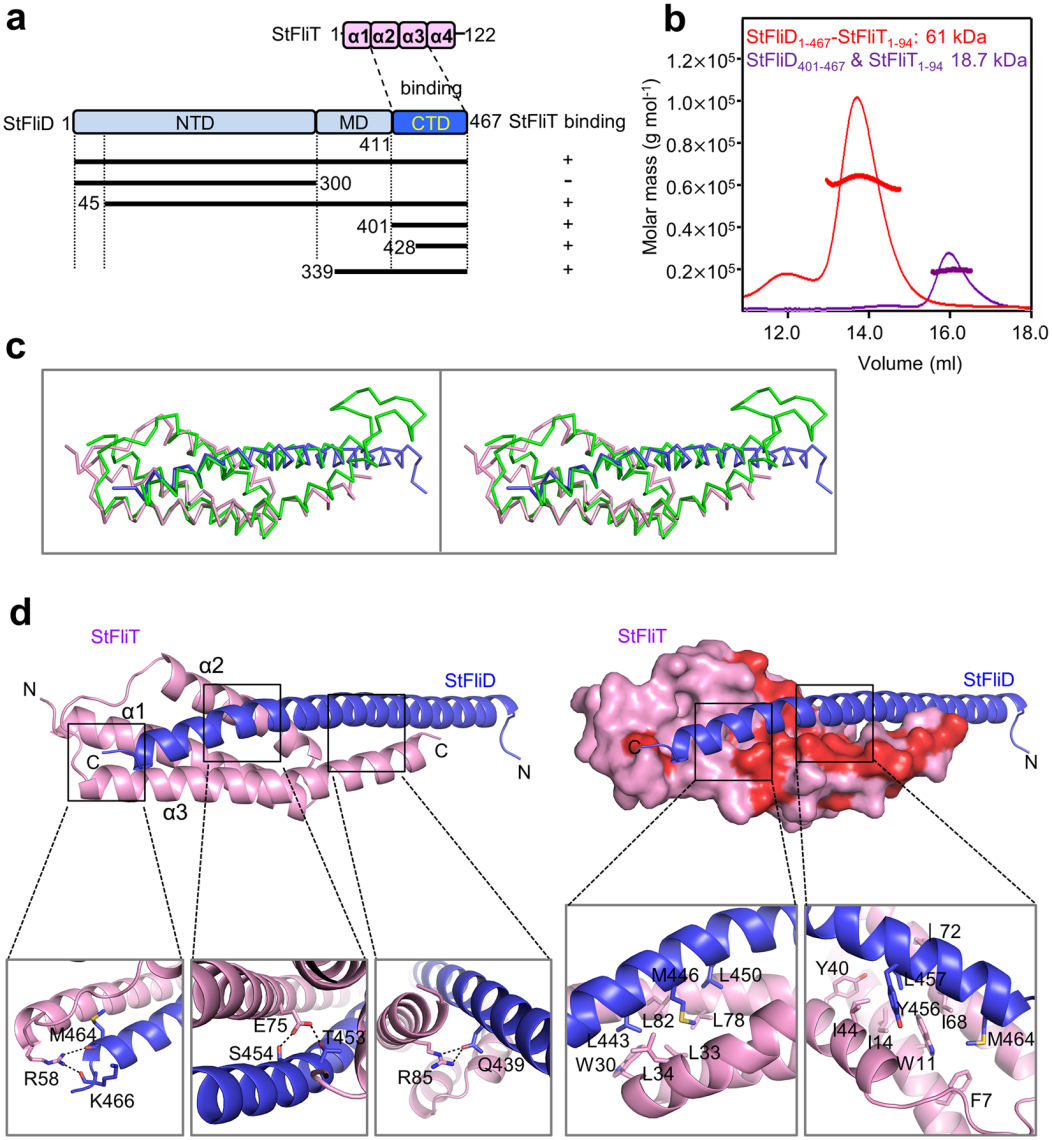
Crystal structure of StFliD-StFliT complex. (**a**) Domain architectures of StFliD and StFliT. (**b**) The StFliD_1–467_-StFliT_1–94_ and StFliD_401–467_-StFliT_1–94_ complexes were analyzed by SEC-MALS. The thick line represents the measured MW. (**c**) Stereo figure superposing crystal and solution structures of StFliD-StFliT complex (PDB codes 5GNA and 5KRW, respectively). Crystal structures of StFliD and StFliT were colored in pink and light blue, respectively, and solution structure of StFliD-StFliT complex was colored in green. (**d**) Ribbon and surface diagrams of the StFliD_401–467_-StFliT_1–94_ complex. Magnified views showing detailed interactions at the StFliD-StFliT interfaces are shown and sequence conservation was mapped on the surface of StFliT in red (100%).

Previously, it was shown that the C-terminal region of FliD from *Salmonella* Typhimurium (StFliD) is responsible for binding to FliT from *S*. Typhimurium (StFliT)^16^. To further analyze the interaction between StFliD and StFliT, various constructs truncating the N- or C-terminal regions of StFliD (StFliD_1–300_, StFliD_45–467_, StFliD_339–467_, StFliD_401–467_, and StFliD_428–467_) were designed and their interactions with StFliT_1–94_ were examined by purifying individual complexes (Fig. 1a and b and Supplementary Fig. S1). Previously, it was reported that the C-terminal segment of StFliT (residues 95–122) does not contribute to its binding to StFliD, but negatively regulates StFliT activity^4^, thus StFliT_1–94_ was used. A stable binary complex of full-length FliD and C-terminus-truncated FliT from *S*. Typhimurium (StFliD and StFliT, respectively) could be reconstituted by coexpressing StFliD_1–467_ and StFliT_1–94_ in *Escherichia coli* cells. The binary complex of StFliD_1–467_ and StFliT_1–94_ was co-eluted from an affinity column and co-migrated on size exclusion columns (Fig. 1a and b). All constructs except StFliD_1–300_ in combination with StFliT_1–94_ were purified and found to be stable (Supplementary Fig. S2A). To analyze the oligomeric states of the StFliD-StFliT complex in solution, we measured the molecular weights (61 kDa and 18.7 kDa) of StFliD_1–467_-StFliT_1–94_ (theoretical molecular weight of 1:1 complex: 61 kDa) and StFliD_401–467_-StFliT_1–94_ (theoretical molecular weight of 1:1 complex: 18.7 kDa) complexes, respectively, using size-exclusion chromatography with multi-angle light scattering (SEC-MALS). The molecular masses measured for both complexes are consistent with a 1:1 complex of StFliD and StFliT in solution (Fig. 1b), which are the same results with the previous reports published in other groups^4, 18^.

### Crystal structure of the StFliD-StFliT complex and its comparison with the solution structure

Recently, the solution structure of StFliT fused with the C-terminal helix of StFliD was solved^16^. To solve the structure of FliT in complex with longer C-terminal helix of FliD, we determined the 2.3-Å resolution crystal structure of StFliD_401–467_ bound to StFliT_1–94_ (Fig. 1c). Nearly the entire sequences of both StFliD_401–467_ and StFliT_1–94_ were found to be ordered, with the exception of the N-terminal flexible loop (11 residues) in StFliD_401–467_ (Supplementary Fig. S2B). StFliD_401–467_ forms a long coil over the entire length (~80 Å) and bound to the concave surface of the StFliT_1–94_ monomer (Fig. 1c and Supplementary Fig. S2). Overall crystal structure of StFliD-StFliT complex was similar to the solution structure^4, 16^ (Fig. 1c). However, the N-terminal region of the C-terminal helix of StFliD was slightly different (Fig. 1c). When both StFliD-StFliT structures were superimposed, the amino terminal parts of StFliD did not overlap well (Fig. 1c). We speculated that the bending of the StFliD C-terminal helix in the solution structure might be induced by the fusion of StFliT with the C-terminal helix of StFliD. The interface between StFliD and StFliT buries several hydrophilic residues (Arg58, Glu75, and Arg85) of StFliT against those of StFliD (Gln439, Thr453, Ser454, Met464, and Lys466) (Fig. 1d). Between these regions, two hydrophobic cores are buried; one consists of five residues (Trp30, Leu33, Leu34, Leu78, and Leu82) of StFliT and three residues (Leu443, Met446, and Leu450) of StFliD (Fig. 1d). The other hydrophobic core consists of seven residues (Phe7, Trp11, Ile14, Tyr40, Ile44, Ile68, and Leu72) of StFliT and three residues (Tyr456, Leu457, and Met464) of StFliD (Fig. 1d).

### Crucial role of Leu443 residue in the StFliD-StFliT complex

**Figure 2:**
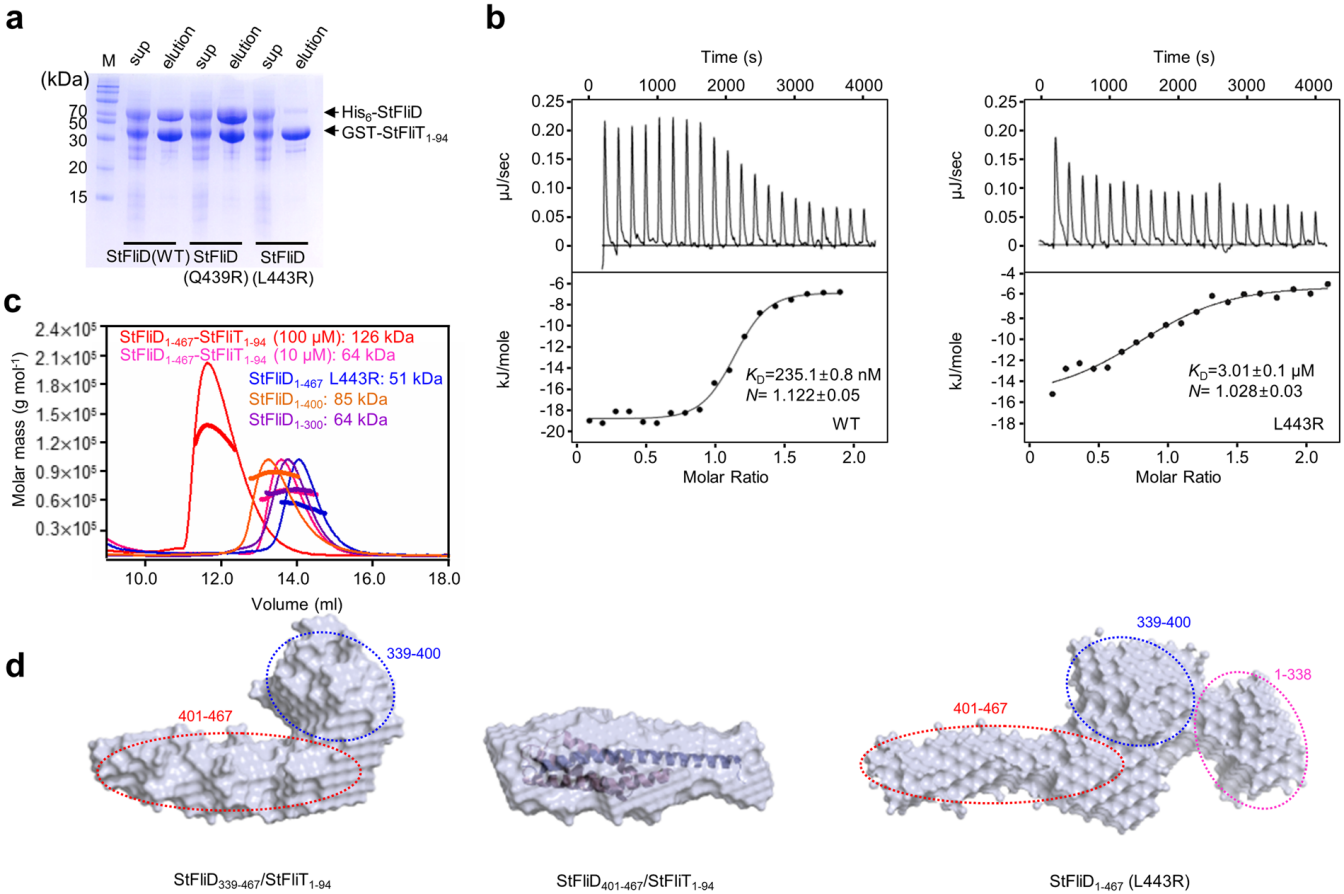
Molecular interactions and SAXS solution structures of various StFliD-StFliT complexes. (**a**) Pull-down assay of his_6_-StFliD (WT, Q439R, and L443R) by GST-StFliT_1–94_. SDS-PAGE gels were visualized using Coomassie blue. (**b**) ITC of StFliT into StFliD solution. The StFliD WT and its mutant (L443R) were titrated against StFliT. (**c**) The StFliD_1–467_-StFliT_1–94_ complex (100 μM in red and 10 μM in pink) and StFliD_1–467_ L443R mutant (100 μM), StFliD_1–300_ (100 μM), and StFliD_1–400_ (100 μM) were analyzed by SEC-MALS. The thick line represents the measured MW. (**d**) SAXS solution structures of StFliD_339–467_-StFliT_1–94_ complex, StFliD_401–467_-StFliT_1–94_ complex, and StFliD_1–467_ L443R mutant. Reconstructed structural models were generated using the *ab initio* shape determination program DAMMIF. Surface renderings of the structural models were generated using PyMOL. To compare the overall shape and dimension of the StFliD_401–467_-StFliT_1–94_ complex, ribbon diagrams of the atomic models were superimposed onto reconstructed dummy atoms models using the program SUPCOMB. Dotted lines (pink, blue, and red) indicate the positions of each domain (residues 1–338, 339–400, and 401–467, respectively).

To evaluate the contributions of the interfacial residues of the StFliD-StFliT complex, the interfacial residues (Gln439 and Leu443) of StFliD were substituted with Arg and their interactions with StFliT were examined using GST pull-down and isothermal titration calorimetry (ITC) (Fig. 2a and b). When the StFliD Gln439, which forms a hydrogen bond with StFliT Arg85, was mutated, the interaction between StFliD and StFliT remained (Fig. 2a). However, when the StFliD Leu443, which is part of the hydrophobic core of the StFliD-StFliT complex, was mutated, the binding affinity between StFliD and StFliT was significantly reduced (Fig. 2a and b), suggesting that the Leu443 residue of StFliD plays a crucial role in StFliD-StFliT complex formation. Full-length StFliD wild-type (WT) strongly bound to StFliT_1–94_ with a dissociation constant of *K*_d_ = 235.1 ± 0.8 nM (Fig. 2b), whereas the L443R mutation reduced the binding affinity (*K*_d_ = 3.01 ± 0.1 μM), suggesting the importance of Leu443 in the direct interaction between StFliD and StFliT in cells.

### SAXS structures of various StFliD-StFliT complexes

We further investigated the molecular details of full-length StFliD and StFliT proteins in an SEC-MALS experiment. The StFliD_1–467_-StFliT_1–94_ complex showed a concentration-dependent quaternary structure in solution. At a concentration of 100 μM, the molecular mass of the StFliD_1–467_-StFliT_1–94_ complex was 126 kDa (theoretical molecular weight of 1:1 complex: 61 kDa), consistent with a 2:2 complex, while the molecular mass was 64 kDa at a concentration of 10 μM, corresponding to a 1:1 complex (Fig. 2c). We hypothesized that the StFliD_1–467_-StFliT_1–94_ heterodimer dimerizes via the N-terminal domains of StFliD, as the C-terminal part of StFliD are engaged in StFliT binding. Consistently, StFliD_1–300_ (100 μM) was a dimer (64 kDa) in solution (theoretical molecular weight of dimer: 65 kDa) (Fig. 2c). To determine the overall conformation of the StFliD_401–467_-StFliT_1–94_ complex in solution, small-angle X-ray scattering (SAXS) data were collected (Fig. 2d and Supplementary Fig. S3–5). The crystal structure of the StFliD_401–467_-StFliT_1–94_ complex was successfully incorporated into the molecular envelope (Fig. 2d and Supplementary Fig. S6A), indicating that the overall conformation of the StFliD_401–467_-StFliT_1–94_ complex is quite rigid in solution. To determine the solution structure of full-length StFliD, we attempted to collect SAXS data collection for the StFliD_1–467_ WT; however, this experiment was unsuccessful. Instead, StFliD_1–467_ (L443R mutant) was used to obtain the *ab initio* structure of the molecular envelope (Fig. 2d and Supplementary Fig. S3–5). The envelope did not show sufficient detail to position the individual domains of StFliD and StFliT. To assign the positions of each domain, the solution structures of various constructs of StFliD were determined (Fig. 2d and Supplementary Fig. S3–5). By comparing the solution structures of the StFliD_339–467_-StFliT_1–94_ and StFliD_401–467_-StFliT_1–94_ complexes, the additional envelope was assigned to the position of StFliD_339–400_ (blue dotted circle) (Fig. 2d). By comparing the solution structures of the StFliD_339–467_-StFliT_1–94_ complex and StFliD_1–467_ (L443R mutant), the additional envelope was assigned to the position of StFliD_1–338_ (pink dotted circle) (Fig. 2d). Collectively, StFliD is tripartite (residues 1–338, 339–400, and 401–467) and StFliT binds only to the C-terminal helix (residues 401–467) of StFliD (Fig. 2d).

### C-terminal region of StFliD is crucial for its pentamer assembly

Interestingly, our study showed that the StFliD L443R mutant exists as a monomer in solution at both high and low concentrations (100 μM and 10 μM, respectively) (Fig. 2c). This indicates that the Leu443 residue of StFliD, which contributes to its interaction with StFliT, plays a crucial role in the pentameric oligomerization of StFliD. These finding suggest that the C-terminal helix of StFliD is critical for the pentameric assembly of StFliD and the binding of StFliT to the C-terminal helix of StFliD can efficiently prevent the pentamer assembly of StFliD. Additionally, the solution structures of the StFliD_45–467_-StFliT_1–94_ heterotetramer and StFliD_1–300_ dimer revealed that the N-terminal domains can dimerize in a head-to-head fashion and both complexes showed a 2-fold symmetrical and elongated molecular shape (Supplementary Fig. S6). In this orientation, the directions of both C-terminal parts of StFliD are opposite, and thus this may not be the orientation of the functional StFliD pentamer at the tip of flagella. These data suggest that the heterotetrameric form of the StFliD-StFliT might exist in the cytosol.

### Physiological effect of the StFliD L443R mutation in *Salmonella* motility

**Figure 3:**
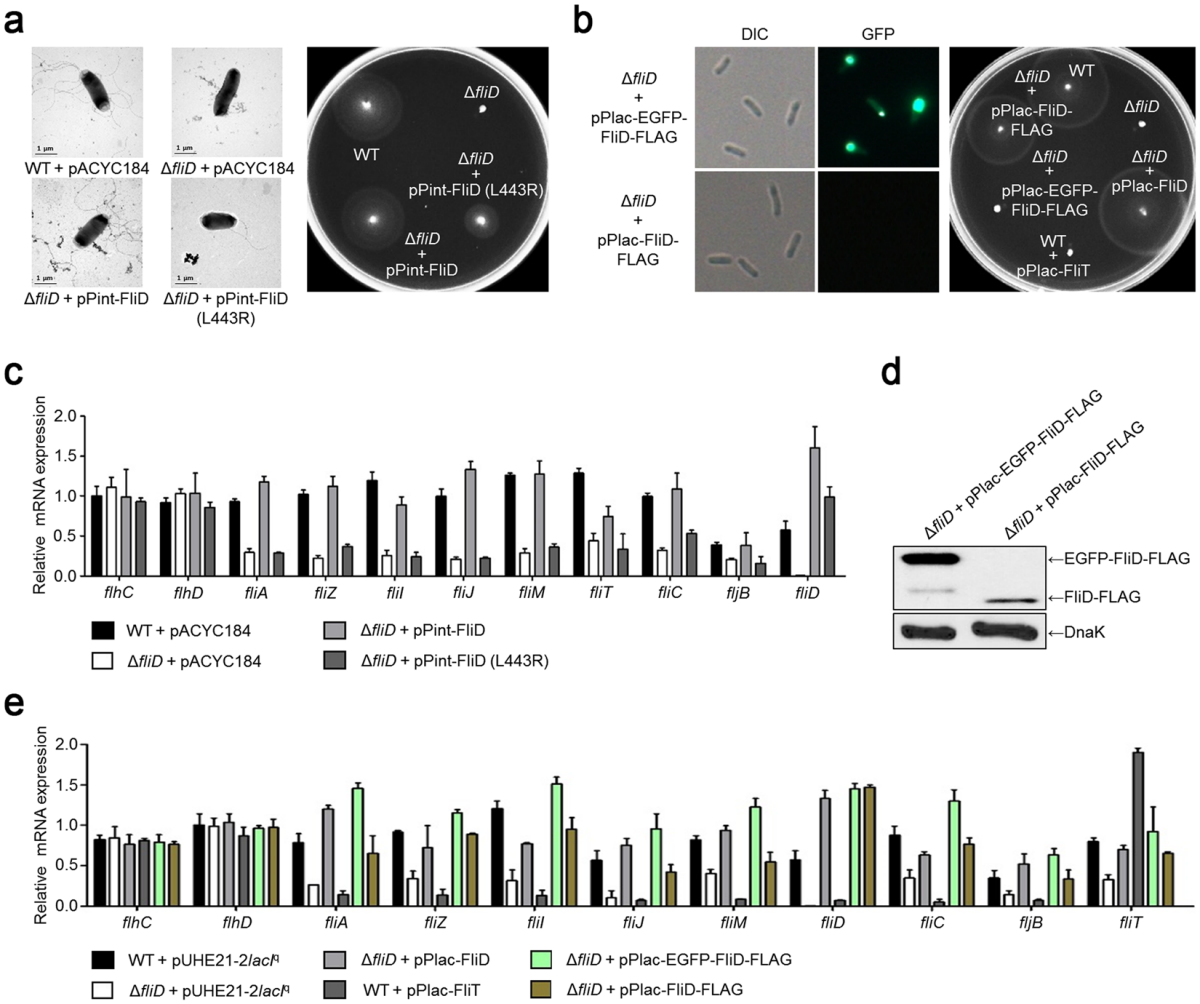
Gene expression patterns of representative genes belonged to three classes of the transcriptional hierarchy. (**a**) Transmission electron micrographs and motile phenotypes of *Salmonella* strains with or without pPint-FliD or pPint-FliD (L443R). Two pACYC184 derivatives (pPint-FliD or pPint-FliD (L443R)) expressed *fliD* or *fliD* (L443R) under its intrinsic promoter, respectively. (**b**) Optical and fluorescent images of *Salmonella* when EGFP-FliD-FLAG protein was expressed in the cell. Right panel shows motile phenotype of *Salmonella* strains with or without pPlac-FliD, pPlac-FliT, pPlac-EGFP-FliD-FLAG, or pPlac-FliD-FLAG, respectively. Four pUHE21-2*lacI* ^q^ derivatives (pPlac-FliD, pPlac-FliT, pPlac-EGFP-FliD-FLAG, or pPlac-FliD-FLAG) overexpressed *fliD*, *fliT*, EGFP-*fliD*-FLAG, or *fliD*-FLAG, respectively, under the *lac* promoter. (**c**) Expression of genes involved in flagella synthesis with or without pPint-FliD or pPint-FliD (L443R) was investigated using qRT-PCR. Wild-type and Δ*fliD* mutant strains with or without pPint-FliD or pPint-FliD (L443R) were grown in LB medium at mid-log phase, and the expression levels of *flhC*, *flhD*, *fliA*, *fliZ*, *fliI*, *fliJ*, *fliM*, *fliT*, *fliC*, *fljB*, and *fliD* relative to that of *gyrB* were determined in each strain. This experiment was performed in triplicate. Two pACYC184 derivatives (pPint-FliD or pPint-FliD (L443R) expressed *fliD* or *fliD* (L443R), respectively, under its intrinsic promoter. (**d**) Comparison of FLAG-tagged StFliD protein levels in the cytosol of Δ*fliD* mutant strain with or without pPlac-EGFP-FliD-FLAG or pPlac-FliD-FLAG (a pUHE21-2*lacI* ^q^ derivative expressing EGFP-FliD-FLAG or FliD-FLAG, respectively) under the *lac* promoter. Flagellins were removed by votexing prior to the western blot analysis in order to analyze StFliD protein levels in the cytosol. (**e**) The effects of StFliD, StFliT, StEGFP-FliD-FLAG or StFliD-FLAG overexpression on the expression of genes involved in flagella synthesis were investigated using qRT-PCR. All procedures were the same as those described above, and four pUHE21-2*lacI* ^q^ derivatives (pPlac-FliD, pPlac-FliT, pPlac-EGFP-FliD-FLAG, or pPlac-FliD-FLAG) overexpressed *fliD*, *fliT*, EGFP-*fliD*-FLAG, or *fliD*-FLAG, respectively, under *lac* promoter.

We further investigated whether the L443R mutation affects its own activity in facilitating the polymerization of *Salmonella* flagellin. It was previously reported that the *Salmonella* Δ*fliD* mutant strain was not motile, but became motile after extracellular addition of purified StFliD protein and subsequent incubation in culture medium^20^. In order to evaluate whether the StFliD L443R mutant can induce the polymerization of flagellin, two purified proteins (StFliD WT or L443R mutant) were added to the Δ*fliD* mutant culture, respectively. Indeed, transmission electron microscopy (TEM) supported that flagellar filaments were synthesized in Δ*fliD* mutant culture in the presence of either the StFliD WT or StFliD L443R mutant (Supplementary Fig. S7). These observations suggest that the StFliD L443R mutant lost its ability to bind to StFliT, but could still synthesize filament at the distal flagellar tip. Thus, we further used the StFliD L443R mutant as a representative mutant to study the interaction between StFliD and StFliT. To investigate the physiological effect of the StFliD-StFliT complex in *Salmonella* motility, we evaluated the behavior of the Δ*fliD* mutant strain in the presence or absence of the pACYC184 vector expressing either the StFliD WT or L443R mutant (pPint-FliD or pPint-FliD (L443R), respectively) under its intrinsic promoter (Fig. 3). Bacterial motility was completely lost in the Δ*fliD* mutant strain, and the introduction of a plasmid expressing StFliD rescued motility to a similar level as in WT (Δ*fliD* + pPint-FliD in Fig. 3a). However, expression of the StFliD L443R mutant resulted in decreased motility as compared to StFliD + (Δ*fliD* + pPint-FliD (L443R) in Fig. 3a). Because the L443R mutation in StFliD significantly lowered its binding to StFliT, unbound StFliT would be free to inhibit FlhD_4_C_2_-dependent flagellar gene expression.

### The role of StFliD Leu443 residue in the negative feedback loop

Previously, it was shown that FliD regulates the ability of FliT to inhibit FlhD_4_C_2_ from activating flagellar gene expression^17^. Thus, StFliD was proposed to act as an anti-regulator of StFliT, suggesting that the secretion of StFliD is essential for the disassembly of the FliD-FliT complex, so that free FliT can bind to the FlhD_4_C_2_ activator^18^. We further studied the negative feedback loop using our StFliD L443R mutant. In order to check whether the StFliD L443R mutation affects the expression of genes involved in flagellar synthesis, qRT-PCR and western blot analysis was carried out using the Δ*fliD* mutant strain transformed with the pACYC184 vector expressing either StFliD WT or L443R mutant under its intrinsic promoter. Representative genes belonging to three classes of the transcriptional hierarchy were selected and monitored (Fig. 3c and Supplementary Fig. S8). We hypothesized that the increased levels of free StFliT proteins caused by StFliD deletion or StFliD L443R mutation had negative effects on the gene expression of flagellar synthesis by binding and inactivating the FlhD_4_C_2_ activator and may reduce the expression of flagellar proteins. Indeed, the overall expression of flagellar genes was decreased in the Δ*fliD* mutant strain (Δ*fliD* + pACYC184) and restored by the expression of StFliD WT (Δ*fliD* + pPint-FliD) (Fig. 3c). The StFliD L443R mutant (Δ*fliD* + pPint-FliD (L443R)), however, could not restore gene expression in contrast to the Δ*fliD* mutant strain expressing exogenous WT StFliD (Fig. 3c). Additionally, western blot analysis revealed decreased levels of flagellar protein in the Δ*fliD* and L443R mutant strains, respectively (Supplementary Fig. S9).

qRT-PCR analysis was also carried out using the Δ*fliD* mutant strain transformed with the pUHE21-2*lacI* ^q^ vector overexpressing either StFliD WT or StFliT WT (Fig. 3b and e). The overall expression of flagellar genes was restored in the Δ*fliD* mutant strain overexpressing StFliD (Δ*fliD* + pPlac-FliD), but significantly decreased by the overexpression of StFliT (WT + pPlac-FliT) (Fig. 3b and e). These results also suggested that large amounts of StFliT negatively affected the gene expression of flagellar synthesis by binding and inactivating the FlhD_4_C_2_ activator. The model in which the secretion of StFliD has the ability to inactivate the FlhD_4_C_2_ activator predicts that the fusion of bulky GFP protein to the N-terminus of StFliD might fail to be exported and might not inactivate the FlhD_4_C_2_ activator. Indeed, the GFP-StFliD fusion increased the amount of GFP-StFliD protein found in the cytosol compared to that of StFliD without GFP (Fig. 3d). Cells expressing the GFP-StFliD protein were defective in motility because of the absence of StFliD cap at the tip of flagellar (Fig. 3b) and the decreased expression of flagellar genes in the Δ*fliD* mutant strain (Δ*fliD*) was restored by the expression of GFP-StFliD (Δ*fliD* + pPlac-EGFP-FliD-FLAG) (Fig. 3e). These results support the previously shown model that the secretion of StFliD is essential for the disassembly of the StFliD-StFliT complex, so that free StFliT can bind to the FlhD_4_C_2_ activator^18^.

### Perturbation of the StFliD-StFliT interaction by L443R mutation affects the lengths of flagellar filaments

**Figure 4:**
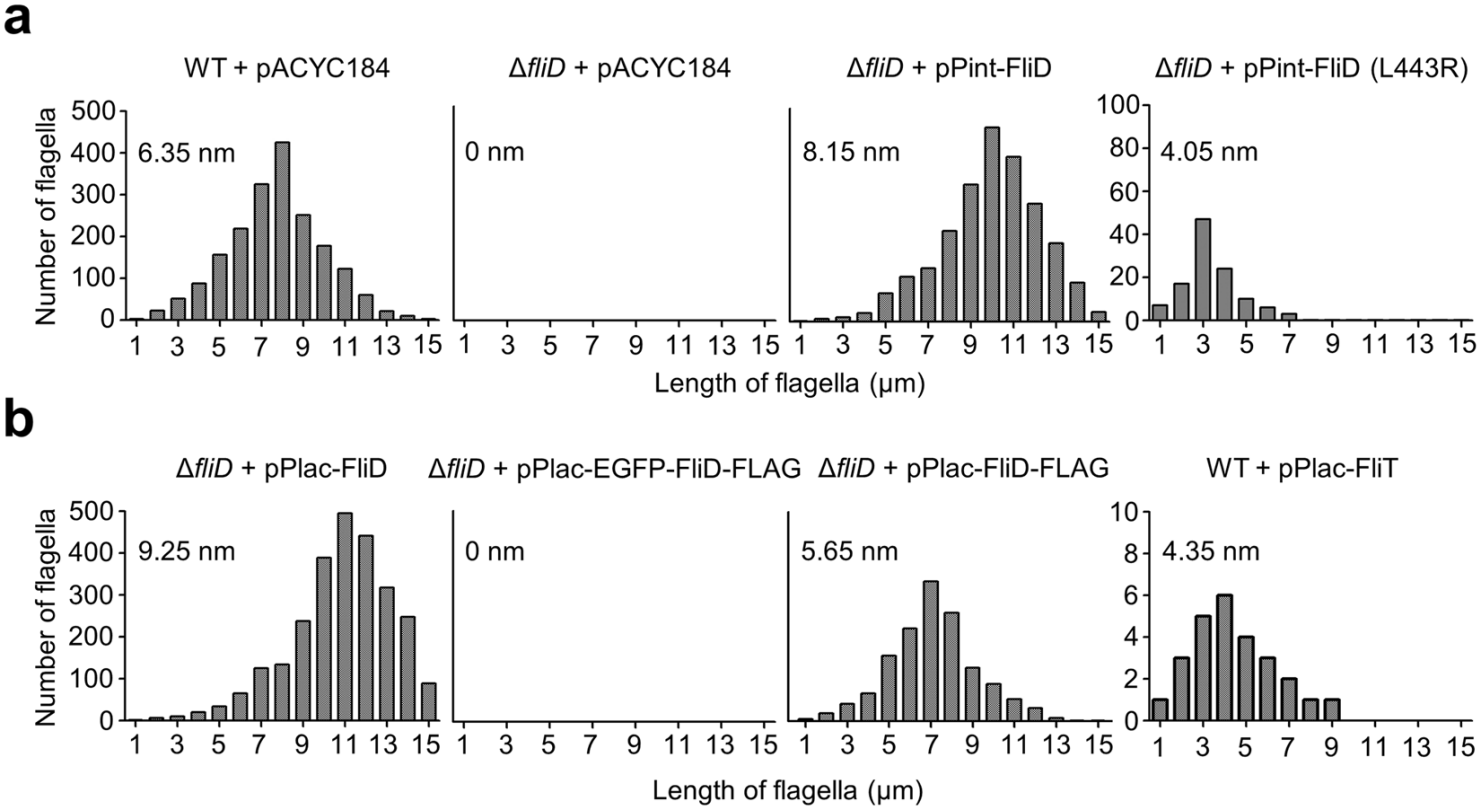
Histogram of filament length or flagella number in *Salmonella* strains. (**a**,**b**) Each strain with or without pPint-FliD, pPint-FliD (L443R), pPlac-FliD, pPlac-FliT, pPlac-EGFP-FliD-FLAG, or pPlac-FliD-FLAG was grown in LB medium to mid-log phase, and filament length or flagella number was measured. pPint-FliD and pPint-FliD (L443R) are pACYC184 derivatives expressing *fliD* or *fliD* (L443R) under the intrinsic promoter, while pPlac-FliD, pPlac-FliT, pPlac-EGFP-FliD-FLAG, and pPlac-FliD-FLAG are pUHE21-2*lacI* ^q^ derivatives overexpressing *fliD*, *fliT*, EGFP-*fliD*-FLAG, or *fliD*-FLAG, respectively, under the *lac* promoter.

We next investigated whether perturbation of the StFliD-StFliT interaction affects the flagellar phenotype. As a representative phenotype of flagellar biogenesis, the lengths of flagellar filaments per cell were monitored (Fig. 4a and b). Interestingly, the lengths of flagella in each cell were significantly reduced in StFliD deletion or L443R mutant strains (Δ*fliD* + pACYC184 or Δ*fliD* + pPint-FliD (L443R), respectively) compared to in the WT *Salmonella* strain (WT + pACYC184) (Fig. 4a), suggesting that normal flagellar biogenesis was impeded because of reduced gene expression of flagellar proteins. The synthesis of flagella was nearly abolished when StFliD was not exported by fusing the bulky GFP to the N-terminus of StFliD (second panel in Fig. 4b). We next hypothesized that the lengths of flagellar were increased and decreased by the overexpression of StFliD and StFliT (Δ*fliD* + pPlac-FliD and WT + pPlac-FliT, respectively). Indeed, the lengths of flagellar slightly increased when StFliD was overexpressed (from 6.35 nm to 9.25 nm) (first panel in Fig. 4b), whereas the synthesis of flagellar was significantly abolished when StFliT was overexpressed (fourth panel in Fig. 4b). These results support the model that the interaction between StFliD and StFliT is crucial for the overall regulation of flagellar gene expression.

### Concluding remarks

The bacterial flagellum is a model system for how very large molecular machines can be assembled in order. According to the morphological development of bacterial flagellum, the expression of flagellar genes is regulated in coordination with assembly. The bacterial flagellum is composed of thousands of protein subunits; thus, the synthesis of unnecessary flagellar proteins may be a burden to bacteria and the negative feedback loop by StFliD secretion may be essential for the life of bacteria^17, 18, 21^. Nevertheless, the feedback loop by the StFliD secretion may not lead to complete repression of flagellar genes. Each cell contains more than one flagellum; thus, the morphological development of each flagellum may differ at different times and basal expression of flagellar genes may be necessary. Consistent with this hypothesis, perturbation of the StFliD-StFliT interaction by expressing the StFliD L443R mutant did not lead to an all-or-nothing response, but basal expression of flagellar genes was detected (Fig. 3c). The feedback loop may control the levels of flagellar protein to adjust the expression of flagellar proteins. Although additional studies are needed to evaluate the molecular mechanism of gene regulation in flagellar biogenesis, the results and model presented here provide a foundation for further analysis.

## Methods

### Protein expression and purification

Constructs comprising residues 401–467 of StFliD and residues 1–94 of StFliT from *S*. Typhimurium were cloned into the pRSF-duet and pGST2 vectors^22^ in frame with an N-terminal six-histidine tag and N-terminal GST tag, respectively. StFliD_401–467_ and StFliT_1–94_ were co-expressed in *E. coli* BL21(DE3) cells induced with 0.5 mM isopropyl-β-D-1-thiogalactopyranoside (IPTG). For cell lysis, cell pellets were resuspended in buffer A (20 mM Tris-HCl, pH 8.0, and 200 mM NaCl) containing 1 mM phenylmethylsulfonyl fluoride. Cells were lysed with a microfluidizer (Microfluidics, Westwood, MA, USA), and the lysed cells were centrifuged at 4,611 g (Vision V506CA rotor) for 30 min at 277 K to pellet the cell debris; the supernatant was applied to a glutathione-Sepharose column (GE Healthcare, Little Chalfont, UK) pre-equilibrated with buffer A. Proteins were eluted with buffer A containing 15 mM reduced glutathione. The eluates were desalted into buffer A, cleaved by TEV protease, and further purified by anion-exchange chromatography with Hitrap Q sepharose column (GE Healthcare). The eluate was further purified by gel filtration on a HiLoad 16/60 Superdex 200 column (GE Healthcare) that had been pre-equilibrated with buffer containing 20 mM Tris-HCl, pH 8.0, and 100 mM NaCl.

Constructs of StFliD_1–467_ (WT, Q439R, and L443R), StFliD_1–300_, StFliD_1–400_, StFliD_45–467_, StFliD_339–467_, and StFliD_428–467_ were cloned into the pRSF-duet vector, respectively. For ITC experiments, each protein of StFliD_1–467_ (WT and L443R, respectively) and StFliT_1–94_ was expressed and purified using a nickel affinity or glutathione-Sepharose column (GE Healthcare), followed by TEV cleavage and gel filtration in buffer containing 20 mM Tris-HCl, pH 8.0, and 200 mM NaCl as described above. The StFliD_1–467_-StFliT_1–94_, StFliD_45–467_-StFliT_1–94_, StFliD_339–467_-StFliT_1–94_, and StFliD_428–467_-StFliT_1–94_ complexes were purified using the same method used to purify the StFliD_401–467_-StFliT_1–94_ complex.

### Crystallization and data collection

Crystals of the StFliD_401–467_-StFliT_1–94_ complex were grown by the sitting-drop vapor diffusion method by mixing equal volumes (1 μL) of each protein solution (~10 mg/mL in buffer containing 20 mM Tris-HCl, pH 8.0, and 100 mM NaCl) and the reservoir solutions. A reservoir solution consisting of 5% PEG400, 10% isopropanol, 0.2 M magnesium chloride, and 0.1 M Tris-HCl, pH 8.0, was used to grow crystals of the StFliD_401–467_-StFliT_1–94_ complex. Crystals of the StFliD_401–467_-StFliT_1–94_ complex reached their maximum size within 1–2 days at 296 K. The crystals were soaked in Paratone-N (Hampton Research, Aliso Viejo, CA, USA) before being flash-frozen in a nitrogen stream at 100 K. Native data for StFliD_401–467_-StFliT_1–94_ were collected at the 5 C beamline of Pohang Accelerator Laboratory (Pohang, South Korea). The raw data were processed and scaled using the program suite *HKL2000* ^23^. Supplementary Table S1 summarizes the statistics of data collection.

### Structure determination and refinement

The structure of the StFliD_401–467_-StFliT_1–94_ complex was solved by the molecular replacement method using the monomer model (chain A, residues 1–94) of StFliT from *S*. Typhimurium (PDB ID: 3A7M). A cross-rotational search followed by a translational search was performed using the *Phaser* program^24^. Subsequent manual model building was carried out using the *COOT* program^25^ and restrained refinement was performed using the *REFMAC5* program^26^. Several rounds of model building, simulated annealing, positional refinement, and individual *B*-factor refinement were performed Supplementary Table S1 lists the refinement statistics. The crystallographic asymmetric unit of the StFliD_401–467_-StFliT_1–94_ complex contains one heterodimer. The refined model includes 193 water molecules, and 100% of the residues are in the most allowed region of the Ramachandran plot. No electron density was observed for residues 401–411 in StFliD. Atomic coordinates and structure factors for the StFliD_401–467_-StFliT_1–94_ complex have been deposited in the Protein Data Bank (PDB ID code 5GNA).

### Size exclusion chromatography with multi-angle light scattering (SEC-MALS)

SEC-MALS experiments were performed using an FPLC system (GE Healthcare) connected to a Wyatt MiniDAWN TREOS MALS instrument and a Wyatt Optilab rEX differential refractometer (Santa Barbara, CA, USA). A Superdex 200 10/300 GL (GE Healthcare) gel-filtration column pre-equilibrated with buffer A was normalized using ovalbumin protein. Proteins were injected (0.5–5 mg) at a flow rate of 0.4 mL/min. Data were analyzed using the Zimm model for fitting static light-scattering data and graphed using EASI graph with a UV peak in ASTRA V software (Wyatt).

### Isothermal titration calorimetry (ITC)

ITC experiments were performed using Affinity ITC instruments (TA Instruments, New Castle, DE, USA) at 298 K. 26 ∫M of StFliD WT and L443R mutant, which were prepared in buffer A were degassed at 295 K prior to measurements. Using a micro-syringe, 2.5 ∫L of 170 ∫M StFliT_1–94_ solution was added at intervals of 200 s to the StFliD (WT and L443R mutant, respectively) solution in the cell with gentle stirring.

### Solution SAXS measurements

Small-angle X-ray scattering (SAXS) measurements were carried out using the 4 C SAXS II beamline of the Pohang Light Source II with 3 GeV power at the Pohang University of Science and Technology, Korea (Supplementary Tables S2 and S3). A light source from an In-vacuum Undulator 20 (IVU20: 1.4 m length, 20 mm period) of the Pohang Light Source II storage ring was focused with a vertical focusing toroidal mirror coated with rhodium and monochromatized with a Si (111) double crystal monochromator, yielding an X-ray beam wavelength of 0.734 Å. The X-ray beam size at the sample stage was 0.1 (V) × 0.3 (H) mm^2^. A two-dimensional (2D) charge-coupled detector (Mar USA, Inc., Evanston, IL, USA) was employed. Sample-to-detector distances of 4.00 m and 1.00 m for SAXS were used. The magnitude of the scattering vector, *q* = (4□/*L*) sin *l*, was 0.1 nm^−1^ < *q* < 6.50 nm^−1^, where 2*l* is the scattering angle and *λ* is the wavelength of the X-ray beam source. The scattering angle was calibrated with polystyrene-b-polyethylene-b-polybutadiene-b-polystyrene block copolymer standard. We used a quartz capillary with an outside diameter of 1.5 mm and wall thickness of 0.01 mm as solution sample cells. All scattering measurements were carried out at 4 °C by using a FP50-HL refrigerated circulator (JULABO, Allentown, PA, USA). SAXS data were collected in six successive frames of 0.1 min each to monitor radiation damage. Measurements of protein solutions were carried out over a wide concentration range (1.0–10.0 mg/mL). Each 2D SAXS pattern was radial-averaged from the beam center and normalized to the transmitted X-ray beam intensity, which was monitored using a scintillation counter placed behind the sample. The scattering of specific buffer solutions was used as the experimental background. The *R*_g,G_ (radius of gyration) values were estimated from the scattering data using Guinier analysis^27^. The molecular mass was calculated from a BSA standard protein. The pair distance distribution *p*(r) function was obtained using the indirect Fourier transform method in the program GNOM^28^.

### Construction of 3D structural models

To reconstruct the molecular shapes, the *ab initio* shape determination program DAMMIF^29^ was used. For each model reconstruction, 10 independent models were selected and the averaged was aligned using the program DAMAVER^30^. The SAXS curves were calculated from the atomic models using the program CRYSOL^31^. To compare the overall shapes and dimensions, ribbon diagrams of the atomic crystal models were superimposed onto the reconstructed dummy atom models using the program SUPCOMB^32^.

### Bacterial strains, plasmids, and culture conditions

*Salmonella* was genetically manipulated using the phage λ Red recombination system^33^ and phage P22-mediated transduction^34^ with *Salmonella enterica* serovar Typhimurium SL1344 as the parent strain. All bacterial strains and plasmids used in this study are listed in Supplementary Table S4. Bacteria were grown aerobically at 310 K in LB medium supplemented with antibiotics. Antibiotics were used at the following concentrations: 50 μg/mL ampicillin, 25 μg/mL chloramphenicol, and 50 μg/mL kanamycin.

### Construction of bacterial strains

The phage λ-derived Red recombination system was used to delete genes in frame or to fuse genes/proteins with peptide tags^33^. To construct SR7031 (Δ*fliD*), the Km^R^ cassette from pKD13 was amplified using primers fliD-del-F and fliD-del-R, and the resultant PCR products were introduced into the SL1344 strain containing the plasmid pKD46. Recombinant bacteria containing the Km^R^ cassette in place of target genes were determined using kanamycin resistance and diagnostic PCR. The Km^R^ cassette was further removed using the plasmid pCP20^21^ as described previously. FlhC, FliA, FliT, or FliC with the FLAG peptide at the C-terminus was also constructed using the phage λ-mediated recombination system^33, 35^. The primers used to construct the bacterial strains are listed in Supplementary Table S5.

### Plasmid construction

To construct pPint-FliD producing FliD under its putative intrinsic promoter, DNA containing the *Salmonella fliD* gene was amplified by PCR using the primers fliD-comple-F and fliD-comple-R, and the PCR products were introduced between the BamHI and SphI sites of pACYC184. Plasmids expressing FliD with L443R substitution were constructed using a QuickChange II site-directed mutagenesis kit (Stratagene, La Jolla, CA, USA) with the primers fliD-L443R-F and fliD-L443R-R for FliD (L443R). pPlac-FliD or pPlac-FliT plasmids producing FliD or FliT under the *lac* promoter, respectively, were constructed. DNA containing the *Salmonella fliD* or *fliT* genes was amplified by PCR using the primers fliD-over-F and fliD-over-R or fliT-over-F and fliT-over-R, and the PCR products were introduced between the BamHI and SalI sites of pUHE21-2l*acI* ^q^. pPlac-EGFP-FliD-FLAG or pPlac-FliD-FLAG plasmids producing EGFP-FliD-FLAG or FliD-FLAG under the *lac* promoter, respectively, were constructed. DNA containing the *Salmonella fliD* gene was amplified by PCR using the primers pET28a-EGFP-FliD-F and pET28a-EGFP-FliD-R, and the PCR products were introduced between the BamHI and HindIII sites of pET28a-EGFP. Afterwards, DNA containing the EGFP-*fliD* gene was amplified by PCR using the primers EGFP-fliD-FLAG-F and EGFP-fliD-FLAG-R, and the PCR products were introduced between SalI and HindIII sites of pUHE21-2*lacI* ^q^ (pPlac-EGFP-FliD-FLAG). DNA containing *Salmonella fliD* gene was also amplified by PCR using fliD-FLAG-F and fliD-FLAG-R, and the PCR products were introduced between SalI and HindIII sites of pUHE21-2*lacI* ^q^ (pPlac-FliD-FLAG). The primers used for plasmid construction are listed in Supplementary Table S5.

### Flagella growth on living cells

Wild-type and Δ*fliD* mutant strains were cultured in LB medium at 310 K with shaking (220 rpm) overnight. Bacterial cultures were inoculated into LB medium again at a 1/100 dilution supplemented with purified FliD or FliD (L443R) proteins (approximately 0.2 mg/mL) and incubated at 310 K for 3 h to the mid-log phase.

### Transmission electron microscopy

*Salmonella* cells were prepared in growth medium that had not been centrifuged, as centrifugation may disrupt fine bacterial structure such as pili, flagella, and capsules. A droplet of the bacterial suspension was placed onto a 50-nm-thick Formvar film that had been transferred onto a copper index TEM grid (Canemco & Marivac, Quebec, Canada). After waiting for 15 min, the remaining solution was wicked away using a piece of filter paper. The samples were then rinsed with 2 mL of 2 mM HEPES buffer, pH 6.8, followed by rinsing with 1 mL of MilliQ water. The samples were then negatively stained with phosphotungstic acid and observed using a LIBRA 120 Energy-Filtering Transmission Electron Microscope (Zeiss, Jena, Germany) at a magnification of ×8000. Bar, 1 μM.

### Motility assay and measurement of filament length

One microliter of an overnight culture was spotted onto the middle of a swim plate (LB medium, 0.3% agar supplemented with antibiotics at the following concentrations: 25 μg/mL chloramphenicol or 50 μg/mL ampicillin, or 0.1 mM IPTG). Plates were allowed to dry for 1 h at room temperature. All plates were incubated at 310 K for 6 h. The flagella images in electron micrographs were analyzed with an image processor (IBAS, Zeise) equipped with a Videoplan program (Zeiss).

### Analysis of EGFP-FliD-FLAG protein in *Salmonella*

Bacterial cells containing pPlac-EGFP-FliD-FLAG plasmid (a pUHE21-2*lacI* ^q^ derivative expressing EGFP-FliD-FLAG under the *lac* promoter) exponentially grown in LB medium with IPTG (0.1 mM) was centrifuged (16000 *g*, 1 min) and resuspended in PBS. The cells were washed twice with PBS buffer and observed by epifluorescence microscopy (DE/Axio Imager A1 microscope, Carl Zeiss, Oberkochen, Germany) with a GFP filter (470/40 nm excitation, 495 nm dichroic, 525/50 nm emission). To compare FLAG-tagged StFliD protein levels in Δ*fliD* mutant strain with or without pPlac-EGFP-FliD-FLAG or pPlac-FliD-FLAG (a pUHE21-2*lacI* ^q^ derivative expressing EGFP-FliD-FLAG or FliD-FLAG, respectively) under the *lac* promoter, protein samples were isolated from each culture grown in LB medium at mid-log phase in the presence of IPTG (0.1 mM) and subjected to western blot analysis. Flagellins were removed by votexing prior to the western blot analysis.

### SDS-PAGE and western blotting analysis

Whole-cell fractions (extracted by sonication or pellets) were dissolved in Laemmli’s sample buffer and boiled for 5 min. The protein samples were loaded onto a 12% SDS-polyacrylamide gel. Proteins were separated on the gel depending on the molecular weights and transferred to a polyvinylidene difluoride membrane. The membrane was blocked with 5% nonfat dry milk in 1× Tris-buffered saline-Tween 20 buffer and probed with anti-FLAG antibody (3:2000 dilution, F1804, Sigma, St. Louis, MO, USA) or anti-DnaK antibody (1:10,000 dilution, ADI-SPA-880, Enzo Life Science, Farmingdale, NY, USA) as primary antibodies. Anti-mouse IgG conjugated to peroxidase (3:5000 dilution, sc-2005, Santa Cruz Biotechnology, Santa Cruz, CA, USA) was used as the secondary antibody in all western blotting experiments. The chemiluminescent signals were developed with a West-Zol plus western blot detection system (Intron Biotechnology, Daejeon, Korea).

### RNA isolation and quantitative real-time RT-PCR

*Salmonella* strains were grown in LB medium and total RNA was isolated at mid-log phage using an RNeasy mini kit (Qiagen, Hilden, Germany). After DNase treatment of the isolated total RNA, cDNA was synthesized by RNA to the cDNA EcoDryPremix (Random Hexamers) (Clontech, Mountain View, CA, USA). cDNA was mixed with 2 × iQ SYBR Green Supermix (Bio-Rad, Hercules, CA, USA) and real-time PCR was performed using the CFX 3.1 (Bio-Rad). The mRNA expression level of the target gene was normalized relative to the *gyrB* (DNA gyrase subunit B) expression level. All primer sets for qRT-PCR used in this study are listed in Supplementary Table S6.

### Data availability

Atomic coordinates and structure factors for the StFliD401–467-StFliT1–94 complex have been deposited in the Protein Data Bank (PDB ID code 5GNA).

## Electronic supplementary material

Supplementary information
