# Supplementary material for: The role of the FliD C-terminal domain in pentamer formation and interaction with FliT
Source: Sci Rep. 2017 Jun 30;7:4418. doi: 10.1038/s41598-017-02664-6 (PMC5493677; doi:10.1038/s41598-017-02664-6)
Supplement: Supplementary file 1 — Supplementary information [file 41598_2017_2664_MOESM1_ESM.pdf]

The role of the FliD C-terminal domain in pentamer formation and interaction with FliT

Hee Jung Kim[1,2,*], Woongjae Yoo[3,*], Kyeong Sik Jin[4], Sangryeol Ryu[3,5] & Hyung Ho Lee[1,†]

[1]Department of Chemistry, College of Natural Sciences, Seoul National University, Seoul 151-742, Korea

[2]Department of Bio & Nano Chemistry, Kookmin University, Seoul 136-702, Korea

[3]Department of Food and Animal Biotechnology, Department of Agricultural Biotechnology, and Research Institute for Agriculture and Life Sciences, Seoul National University, Seoul 08826, Korea

[4]Pohang Accelerator Laboratory, Pohang University of Science and Technology, 80 Jigokro-127-beongil, Nam-Gu, Pohang, Kyungbuk 37673, Korea

[5]Center for Food and Bioconvergence, Seoul National University, Seoul 08826, Korea

[*]These authors contributed equally to this work.

[†]Corresponding author:

Professor Hyung Ho Lee, Department of Chemistry, College of Natural Sciences, Seoul National University, Seoul 08826, Korea

Supplementary figures

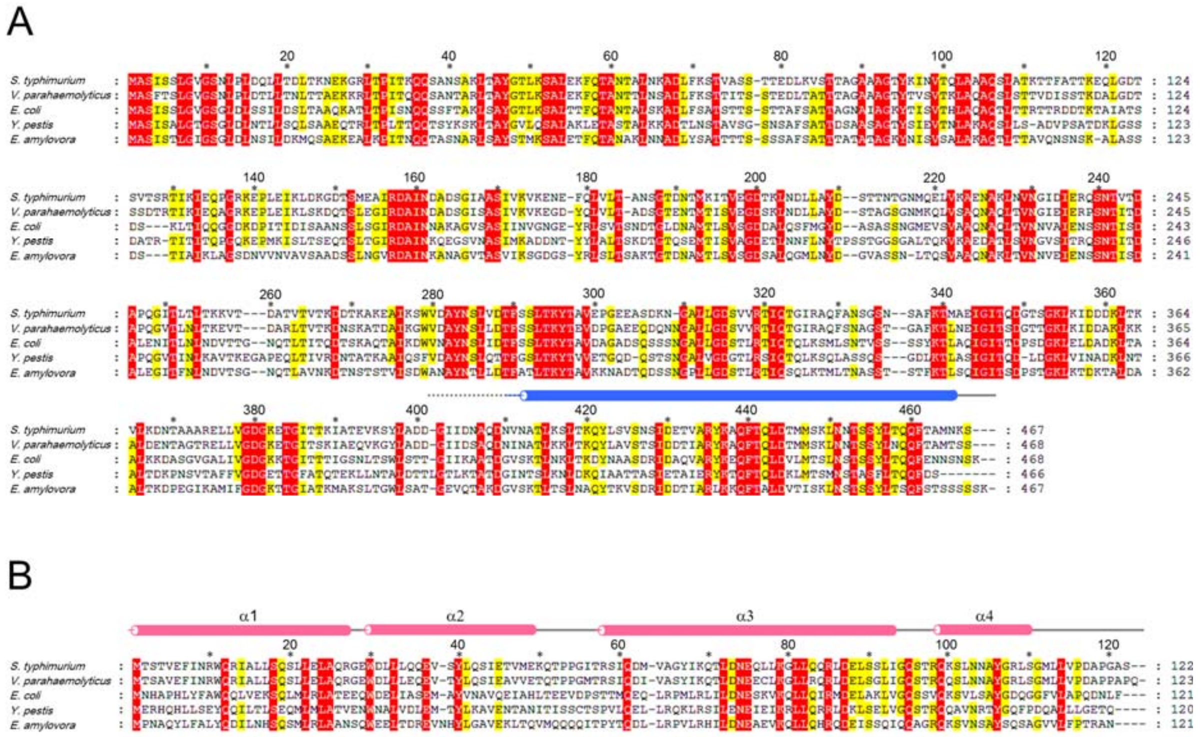

Figure S1. Sequence alignments of StFliD and StFliT. (**A**) Multi-alignment of *S.* Typhimurium FliD (UniProtKB/Swiss-Prot accession number P16328) against FliD from *Vibrio parahaemolyticus* (UniProtKB/Swiss-Prot accession number A0A0M3E723), *E. coli* (UniProtKB/Swiss-Prot accession number P24216), *Yersinia pestis* (UniProtKB/Swiss-Prot accession number Q7CHZ9), and *Erwinia amylovora* (UniProtKB/Swiss-Prot accession number D4HVZ3). Secondary structure elements were assigned by PyMOL (The PyMOL Molecular Graphics System, http://www.pymol.org) and every tenth residue is marked by a black star. Strictly (100%) and semi-conserved (above 80%) residues are highlighted in red and yellow, respectively. Cylinders above the sequences denote α-helices. A dotted line denotes disordered regions. (**B**) Multi-alignment of *S.* Typhimurium FliT (UniProtKB/Swiss-Prot accession number

P0A1N2) against FliT from *V. parahaemolyticus* (UniProtKB/Swiss-Prot accession number A0A0M3E5B6), *E. coli* (UniProtKB/Swiss-Prot accession number P0ABY2), *Y. pestis* (UniProtKB/Swiss-Prot accession number Q8D0B5), and *E. amylovora* (UniProtKB/Swiss-Prot accession number D4HVZ5), respectively.

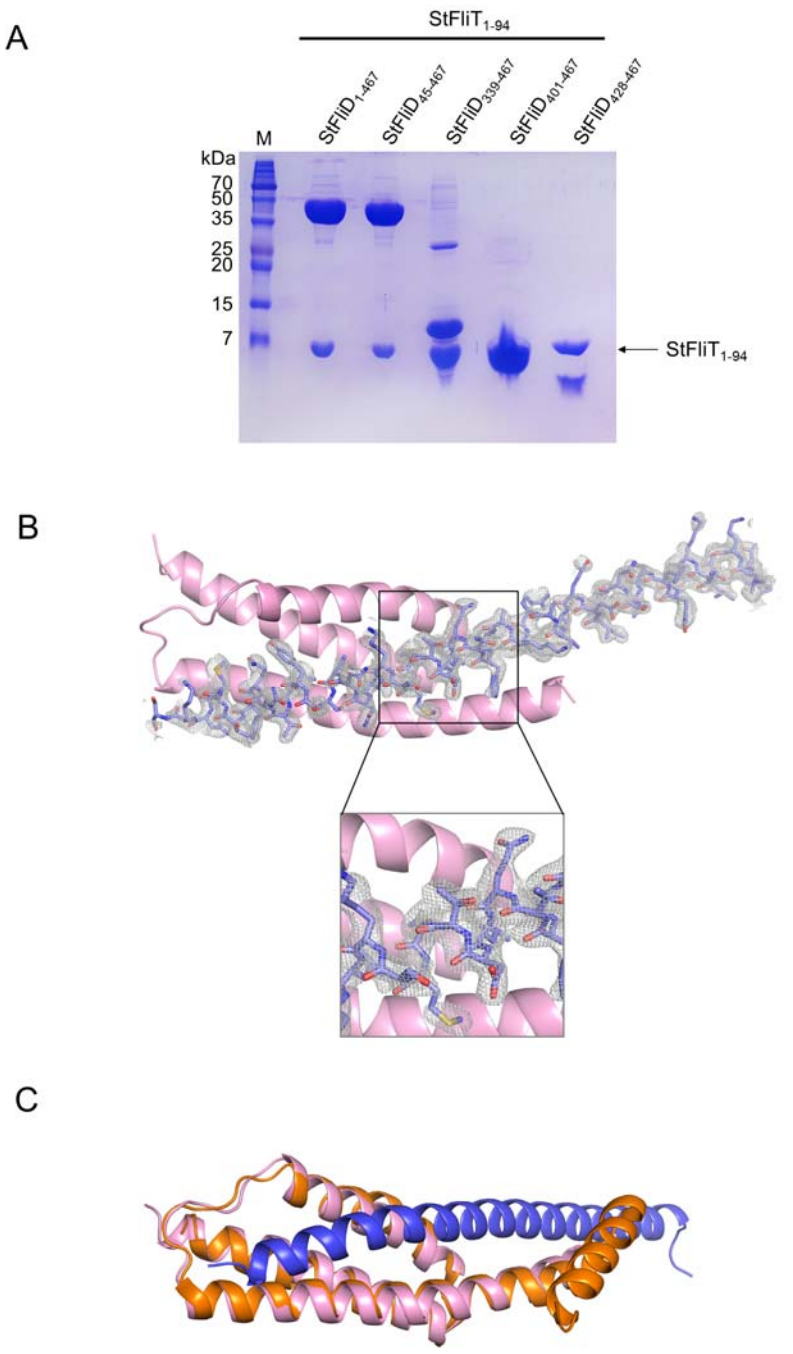

Figure S2. Co-purification of various StFliD-StFliT complexes and omit map of the $StFliD_{401\text{-}467}$-$StFliT_{1\text{-}94}$ complex. (**A**) SDS-PAGE of co-purified $StFliD_{1\text{-}467}$-$StFliT_{1\text{-}94}$, $StFliD_{45\text{-}467}$-$StFliT_{1\text{-}94}$, $StFliD_{339\text{-}467}$-$StFliT_{1\text{-}94}$, $StFliD_{401\text{-}467}$-$StFliT_{1\text{-}94}$, and $StFliD_{428\text{-}467}$-$StFliT_{1\text{-}94}$ complexes. SDS-PAGE gels were visualized using Coomassie blue. (**B**) A composite simulated

annealed omit map (1.5 sigma) for $StFliD_{401-467}$ of the $StFliD_{401-467}$-$StFliT_{1-94}$ complex. (**C**) Superposition of crystal structures of StFliD-StFliT complex (PDB code 5GNA, pink and light blue, respectively,) and StFliT alone (orange, PDB code 3A7M).

A

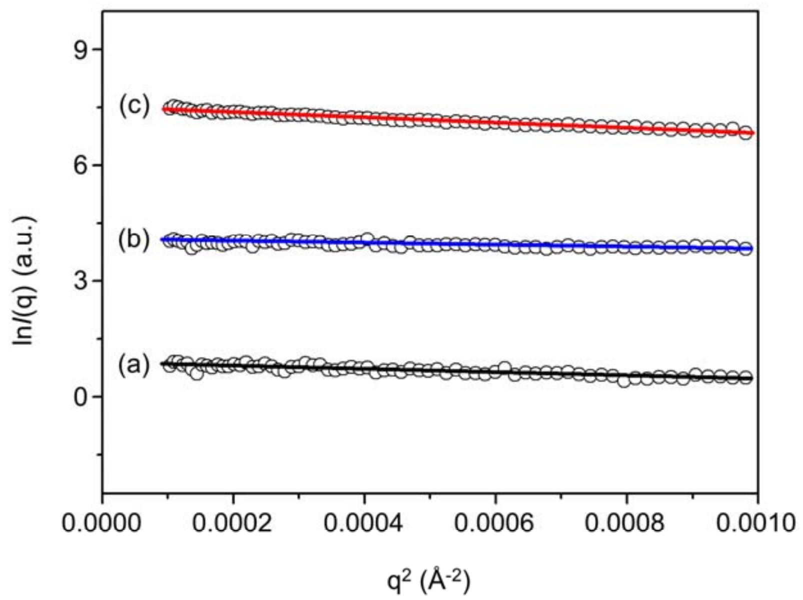

B

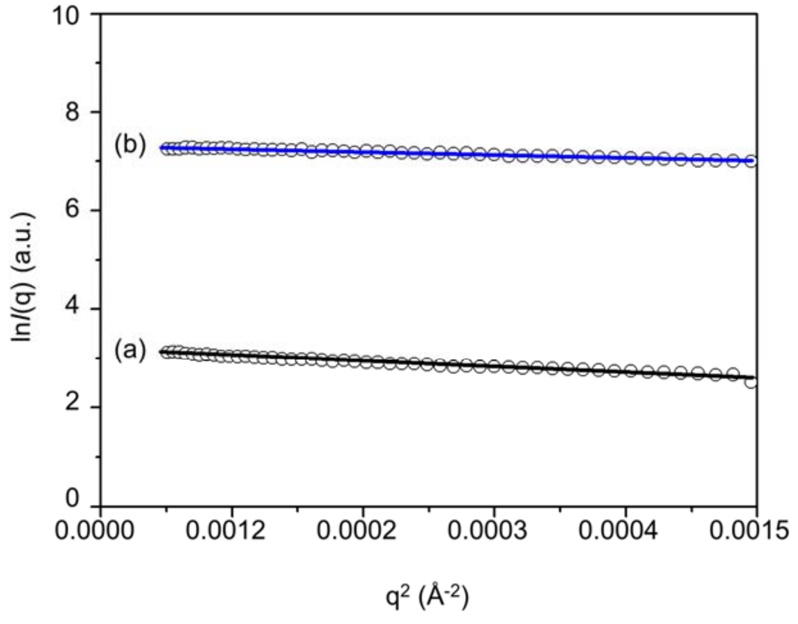

Figure S3. Guinier plots of X-ray scattering profiles of StFliD-StFliT proteins in aqueous solution. (**A**) The $StFliD_{339\text{-}467}$-$StFliT_{1\text{-}94}$ complex, $StFliD_{401\text{-}467}$-$StFliT_{1\text{-}94}$ complex, and $StFliD_{1\text{-}}$

$_{467}$ L443R mutant are shown in (a), (b), and (c), respectively. The straight lines were obtained from the linear regression of the scattering data in the q^2 region. For clarity, each plot is shifted along the ln $I(q)$ axis. (**B**) The StFliD$_{45\text{-}467}$-StFliT$_{1\text{-}94}$ complex and StFliD$_{1\text{-}300}$ are shown in (a) and (b), respectively. Straight lines were obtained from linear regression of the scattering data in the q^2 region. For clarity, each plot is shifted along the ln $I(q)$ axis.

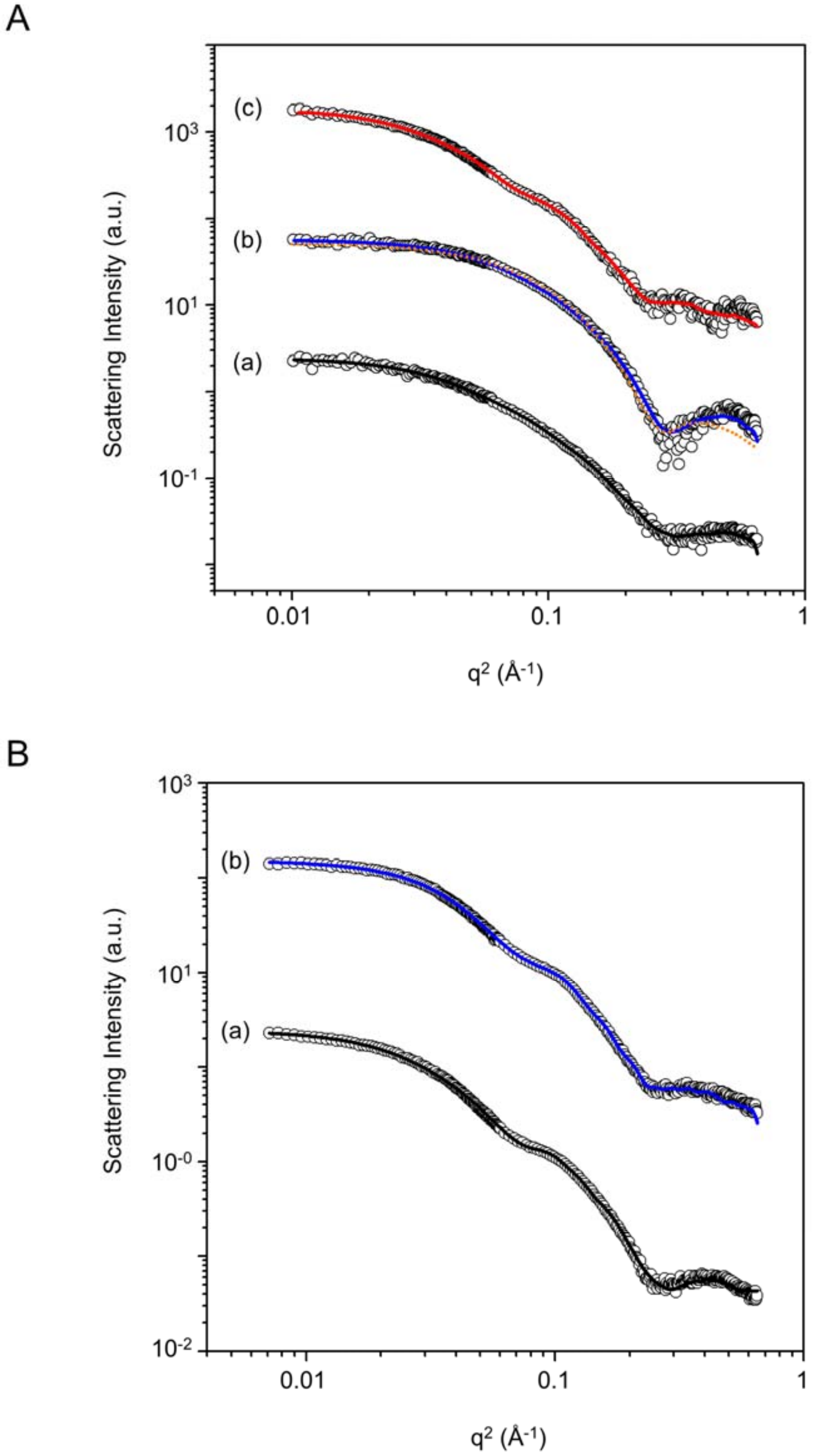

Figure S4. X-ray scattering profiles of StFliD-StFliT proteins in solution. (**A**) The StFliD$_{339-467}$-StFliT$_{1-94}$ complex, StFliD$_{401-467}$-StFliT$_{1-94}$ complex, and StFliD$_{1-467}$ L443R mutant are shown

in (a), (b), and (c), respectively. The open symbols indicate experimental data and the solid lines indicate X-ray scattering profiles obtained from the dummy atom models with the lowest χ^2 = 0.021–0.025 values obtained using the program DAMMIF. The dashed line is the theoretical SAXS curve calculated from the crystal structure of $StFliD_{412-467}$-$StFliT_{1-94}$ protein using the program CRYSOL (χ^2 = 0.130). For clarity, each plot is shifted along the log *I(q)* axis. (**B**) The $StFliD_{45-467}$-$StFliT_{1-94}$ complex and the $StFliD_{1-300}$ are shown in (a) and (b), respectively. The open symbols indicate experimental data and the solid lines indicate X-ray scattering profiles obtained from the dummy atom models with the lowest χ^2 = 0.052–0.160 values determined using the program DAMMIF. For clarity, each plot is shifted along the log *I(q)* axis.

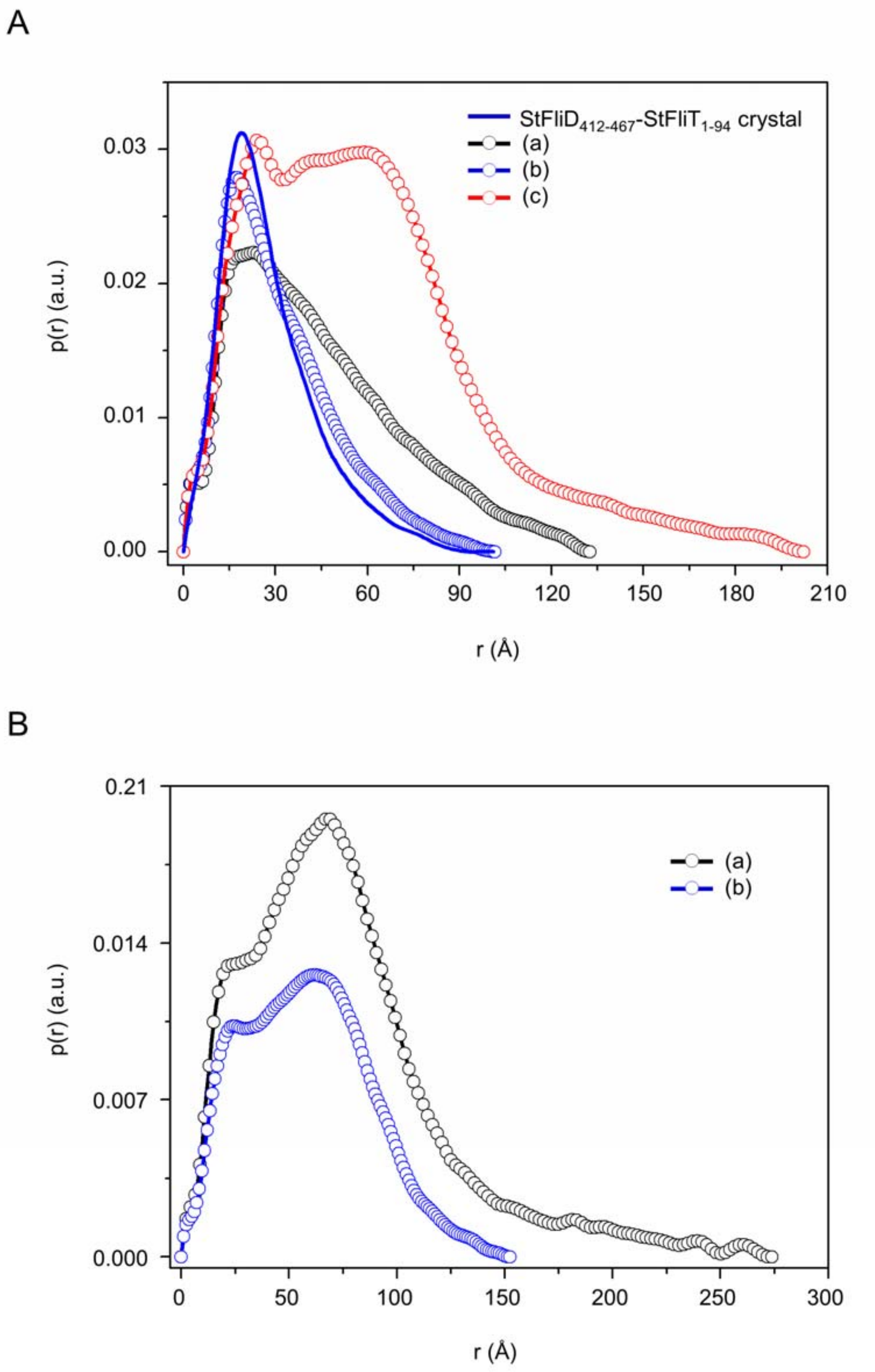

Figure S5. Pair distance distribution *p*(*r*) functions of StFliD-StFliT proteins in aqueous solution based on analysis of experimental SAXS data using the program GNOM. (**A**) The

$StFliD_{339\text{-}467}$-$StFliT_{1\text{-}94}$ complex, $StFliD_{401\text{-}467}$-$StFliT_{1\text{-}94}$ complex, and $StFliD_{1\text{-}467}$ L443R mutant are shown in (a), (b), and (c), respectively. The areas under the curves were normalized to the molecular weights. (**B**) The $StFliD_{45\text{-}467}$-$StFliT_{1\text{-}94}$ complex and $StFliD_{1\text{-}300}$ are shown in (a) and (b), respectively. The areas under the curves were normalized to the molecular weights.

A

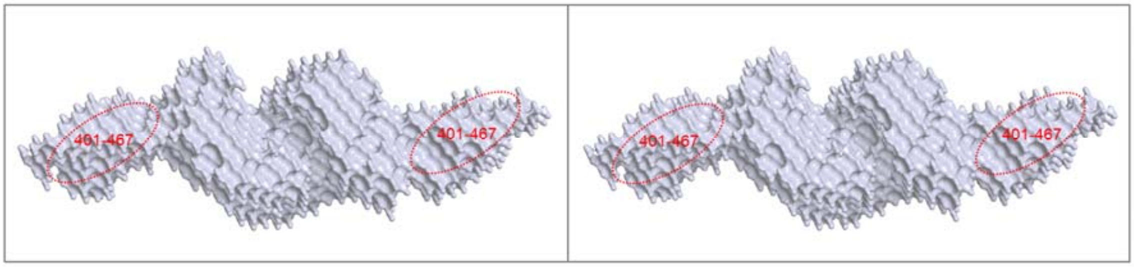

$StFliD_{45\text{-}467}$-$StFliT_{1\text{-}94}$

B

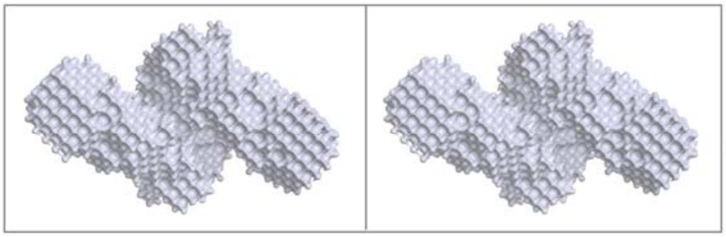

$StFliD_{1\text{-}300}$

Figure S6. SAXS solution structures of the $StFliD_{45\text{-}467}$-$StFliT_{1\text{-}94}$ complex and $StFliD_{1\text{-}300}$. (**A**, **B**) Stereo reconstructed structural models of the $StFliD_{45\text{-}467}$-$StFliT_{1\text{-}94}$ complex and $StFliD_{1\text{-}300}$ were generated using the *ab initio* shape determination program DAMMIF. Surface renderings of the structural models were generated using the program PyMOL. Dotted lines indicate the positions of each domain.

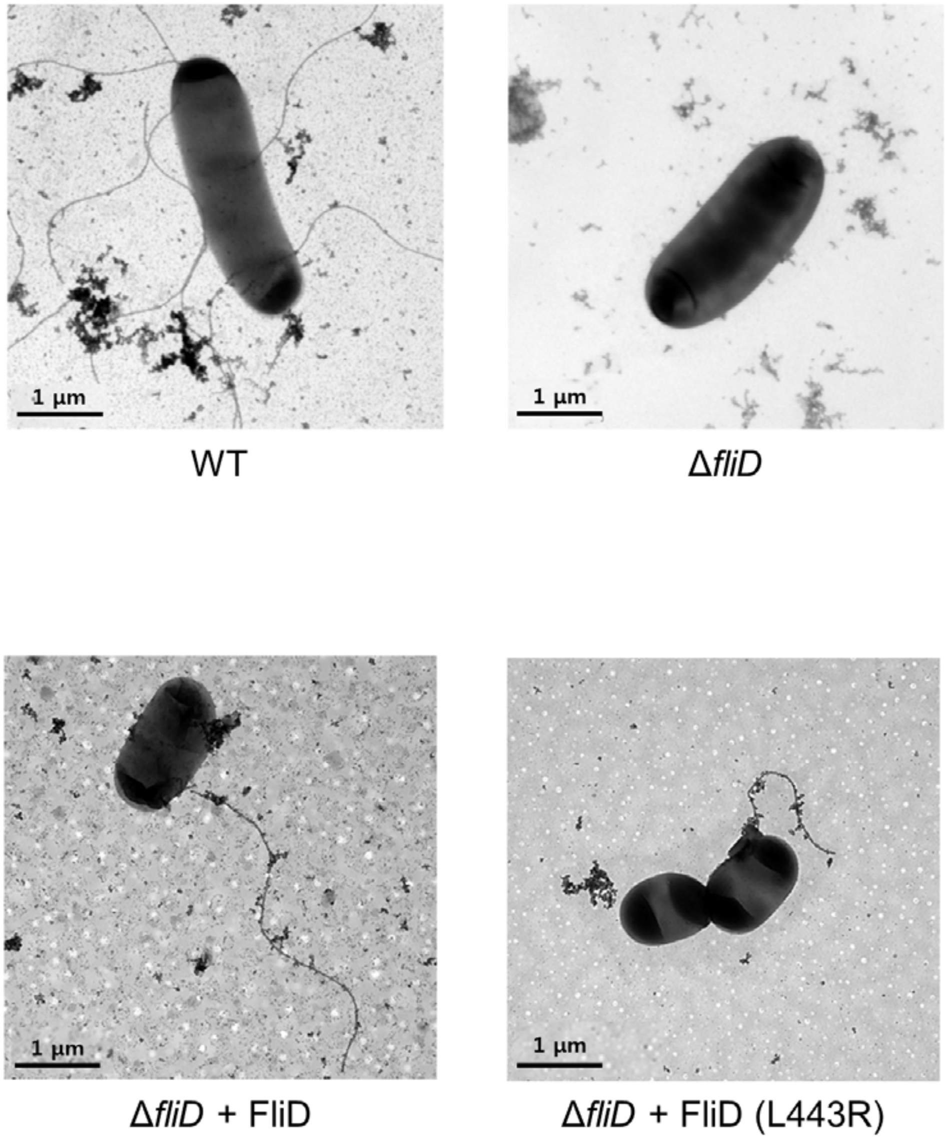

Figure S7. Flagellar regeneration activity of purified $StFliD_{1\text{-}467}$ WT and $StFliD_{1\text{-}467}$ L443R proteins. Cells grown in LB broth were stained negatively with phosphotungstic acid and observed by TEM. Purified $StFliD_{1\text{-}467}$ WT and $StFliD_{1\text{-}467}$ L443R proteins were added to the culture of the Δ*fliD* mutant strain to facilitate flagellar regeneration. Micrographs were acquired at a magnification of ×8000. Bar, 1 μM.

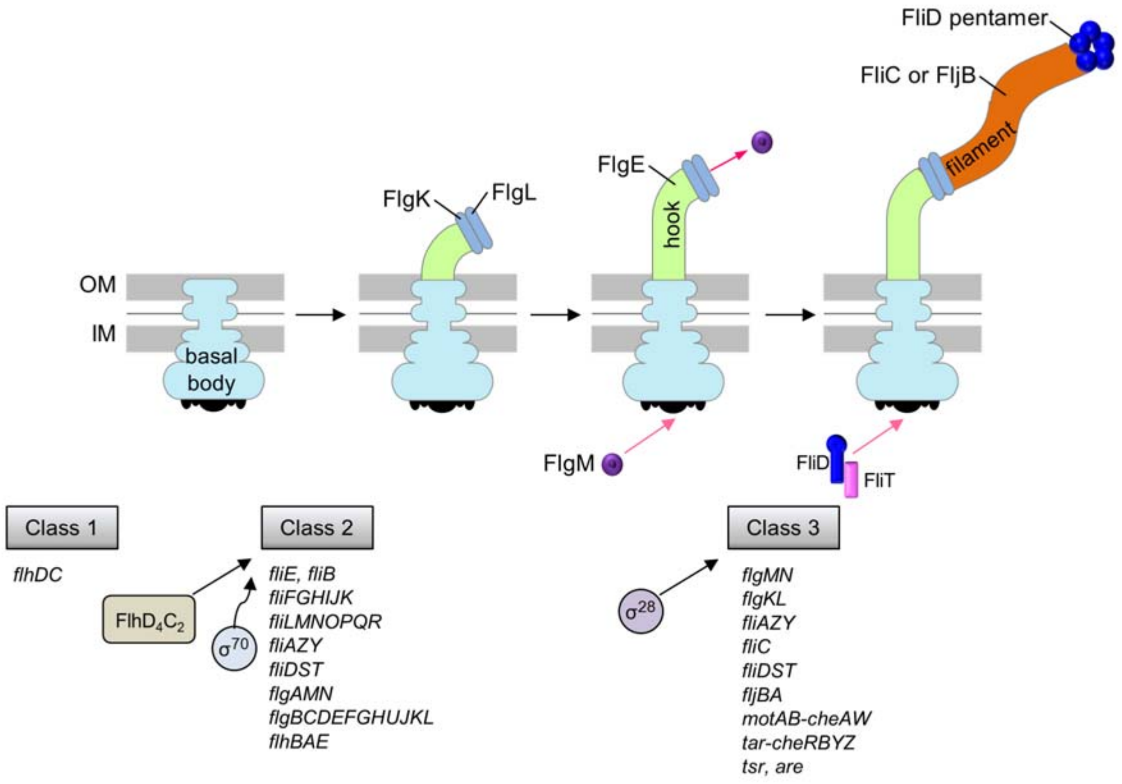

Figure S8. Schematic diagram of flagellar transcriptional hierarchy.

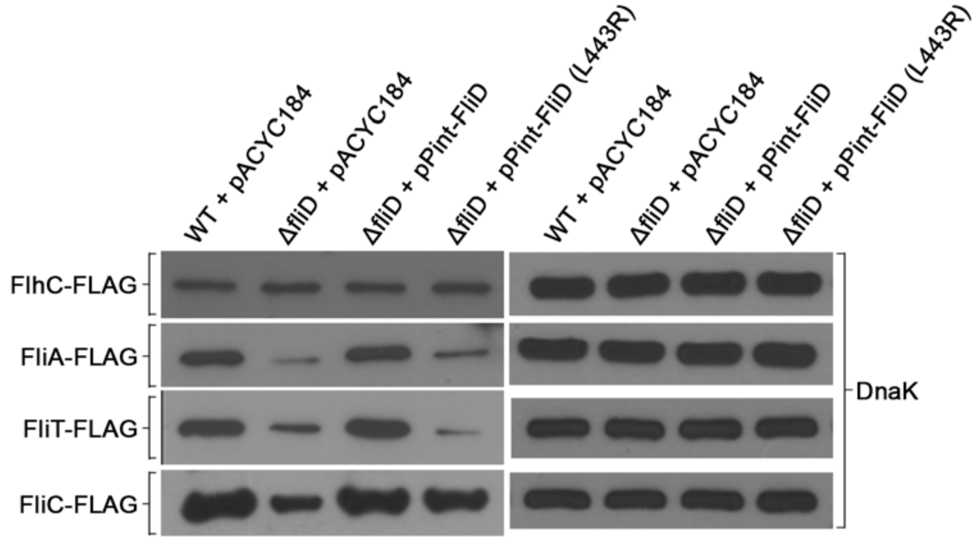

Figure S9. Western blot analysis using Δ*fliD* mutant strain transformed with pACYC184 vector expressing either StFliD WT or L443R mutant. Comparison of FLAG-tagged FlhC, FliA, FliT, and FliC protein levels between wild-type and Δ*fliD* mutant strain with or without pPint-FliD or pPint-FliD (L443R) (a pACYC184 derivative expressing *fliD* or *fliD* (L443R), respectively) under its intrinsic promoter. Protein samples were isolated from each culture grown in LB medium at mid-log phase and subjected to western blot analysis. The expression the FlhC, FliA, FliT, and FliC proteins was assessed using an anti-FLAG antibody. In all experiments, equivalent amounts of total protein were loaded into each lane for SDS-PAGE and DnaK levels were measured in parallel to verify equivalent total protein loading between lanes.

Supplementary tables

Table S1. Statistics for data collection and refinement.

Data set	$StFliD_{401\text{-}467}$-$StFliT_{1\text{-}94}$ complex
A. Data collection statistics	
X-ray source	PLS BL-5C
X-ray wavelength (Å)	0.97960
Space group	$P4_322$
a, *b*, *c* (Å)	50.4, 50.4, 184.6
Resolution range (Å)	50-2.3
Total / unique reflections	307,692 / 11,357
Completeness (%)	100.0 (100.0)[a]
Average $I/\sigma(I)$	87.6 (17.5)[a]
R_{merge}[b] (%)	8.4 (40.9)[a]
B. Model refinement statistics	
Resolution range (Å)	30-2.3
R_{work} / R_{free}[c] (%)	21.4 / 23.0
Number / average *B*-factor (Å^2)	
Protein nonhydrogen atoms	1208 / 51.2
Water oxygen atoms	193 / 60.6
R.m.s. deviations from ideal geometry	
Bond lengths (Å)	0.006
Bond angles (°)	0.922
Protein-geometry analysis	
Ramachandran favored (%)	100.0 (148/148)
Ramachandran allowed (%)	0.0 (0/148)
Ramachandran outliers (%)	0.0 (0/148)

Footnotes for table S1

[a] Values in parentheses refer to the highest resolution shell (2.34-2.30 Å).

[b] $R_{merge} = \Sigma_{hkl}\Sigma_i \mid I_i(hkl) - <I(hkl)> \mid / \Sigma_{hkl}\Sigma_i I_i(hkl)_i$, where $I(hkl)$ is the intensity of reflection *hkl*, Σ_{hkl} is the sum over all reflections, and Σ_i is the sum over i measurements of reflection *hkl*.

[c] $R = \Sigma_{hkl} \mid |F_{obs}| - |F_{calc}| \mid / \Sigma_{hkl} |F_{obs}|$, where R_{free} was calculated for a randomly chosen 10% of reflections, which were not used for structure refinement and R_{work} was calculated for the remaining.

Table S2. Structural parameters obtained from the SAXS data of the StFliD$_{339-467}$-StFliT$_{1-94}$ complex, the StFliD$_{401-467}$-StFliT$_{1-94}$ complex, and StFliD$_{1-467}$ L443R mutant in solution.

Sample	$R_{g,G}$[a] (Å)	$R_{g,p(r)}$[b] (Å)	D_{max}[c] (Å)	$MM_{calculated}$[d] (kDa)	MM_{SAXS}[e] (kDa)	Vp[f] (Å^3)
Crystal structure [g]	21.59 ± 0.07	22.69 ± 0.01	98	(18.7)	-	24060[h]
StFliD$_{339-467}$/StFliT$_{1-94}$ complex	34.24 ± 1.51	36.75 ± 2.11	133	25.3	28.4	44607
StFliD$_{401-467}$-StFliT$_{1-94}$ complex	24.10 ± 0.78	25.64 ± 1.08	102	18.7	18.4	26009
StFliD$_{1-467}$ L443R	46.29 ± 2.02	49.35 ± 3.68	203	49.8	50.0	101264

Footnotes for table S2

[a]$R_{g,G}$ (radius of gyration) was obtained from the scattering data by the Guinier analysis.

[b]$R_{g,p(r)}$ (radius of gyration) was obtained from the $p(r)$ function by the program GNOM.

[c]D_{max} (maximum dimension) was obtained from the $p(r)$ function by the program GNOM.

[d]$MM_{calculated}$ (molecular mass) was obtained from the amino acid sequence of protein.

[e]MM_{SAXS} (molecular mass) was estimated from a BSA standard protein.

[f]V_p(Porod volume) was determined from the program PRIMUS.

[g]Crystal structure of StFliD$_{401-467}$-StFliT$_{1-94}$ complex.

[h]Envelope volume was determined from the atomic crystal structure by the program CRYSOL.

Table S3. Structural parameters obtained from the SAXS data of the $StFliD_{45\text{-}467}$-$StFliT_{1\text{-}94}$ complex and $StFliD_{1\text{-}300}$ in solution.

Sample	$R_{g,G}$[a] (Å)	$R_{g,p(r)}$[b] (Å)	D_{max}[c] (Å)	$MM_{calculated}$[d] (kDa)	MM_{SAXS}[e] (kDa)	Vp[f] ($Å^3$)
$StFliD_{45\text{-}467}$/$StFliT_{1\text{-}94}$ complex	55.70 ± 0.99	61.96 ± 2.50	275	56.4	112.5	211931
$StFliD_{1\text{-}300}$	44.26 ± 0.72	45.86 ± 0.50	153	31.9	62.1	125360

Footnotes for table S3

[a]$R_{g,G}$ (radius of gyration) was obtained from the scattering data by the Guinier analysis.

[b]$R_{g,p(r)}$ (radius of gyration) was obtained from the $p(r)$ function by the program GNOM.

[c]D_{max} (maximum dimension) was obtained from the $p(r)$ function by the program GNOM.

[d]$MM_{calculated}$ (molecular mass) was obtained from the amino acid sequence of protein.

[e]MM_{SAXS} (molecular mass) was estimated from a BSA standard protein.

[f]V_p(Porod volume) was determined from the program PRIMUS.

[g]Envelope volume was determined from the atomic crystal structure by the program CRYSOL.

Table S4. Bacterial strains and plasmids used in this study.

**Strains**	**Description**	**Reference or source**
Salmonella enterica **serovar Typhimurium**		
SL1344	Wild type, Sm^R	(*1*)
SR7031	Δ*fliD*	This study
SR7032	FlhC-FLAG	This study
SR7033	FlhC-FLAG, Δ*fliD*	This study
SR7034	FliA-FLAG	This study
SR7035	FliA-FLAG, Δ*fliD*	This study
SR7036	FliT-FLAG	This study
SR7037	FliT-FLAG, Δ*fliD*	This study
SR7038	FliC-FLAG	This study
SR7039	FliC-FLAG, Δ*fliD*	This study
E. coli		
DH5α	*gyrA96 recA1 relA1 endA1 thi-1 hsdR17 glnV44 deoR Δ(lacZYA-argF)U169* [Φ80d Δ(*lacZ*)*M15*]	(*2*)
**Plasmids**		
pKD46	Ap^R P_{BAD}-*gam-beta-exo oriR101* $repA101^{ts}$	(3)
pKD13	Ap^R FRT Km^R FRT PS1 PS4 *oriR6Kγ*	(3)
pCP20	Ap^R Cm^R *cI*857 λP_R*flp* $oripSC101^{ts}$	(3)
pACYC184	Tet^R Cm^R p15A *ori*	(4)
pUHE21-2$lacI^q$	rep_{pMB1} Ap^R $lacI^q$	(5)
pT25-*fliD*	pKT25-*fliD*	This study
pT25-*fliD* (L443R)	pKT25-*fliD* (L443R)	This study
pT18-*fliT*	pUT18C-*fliT*	This study
pPint-FliD	pACYC184-*fliD*	This study
pPint-FliD (L443R)	pACYC184-*fliD* (L443R)	This study
pPlac-FliD	pUHE21-2$lacI^q$-*fliD*	This study
pPlac-FliT	pUHE21-2$lacI^q$-*fliT*	This study
pEGFP-FliD	pET28a-EGFP-FliD	(6)

pPlac-EGFP-FliD-FLAG	pUHE21-2*lacI*^{q}-EGFP-*fliD*-FLAG	This study
pPlac-FliD-FLAG	pUHE21-2*lacI*^{q}-*fliD*-FLAG	This study

Table S5. Primers used for the construction of bacterial strains and plasmids.

Primers	Sequences (5' to 3')
fliD-del-F	TAC CAA ACA GCA GAG CGC GAA TTC GGC AAA GCT AAC CGC CTG TAG GCT GGA GCT GCT TCG
fliD-del-R	CAT CAT CAA TCT TCA GTT TGC CGG AAG TCC CAT CCT GGG TAT TCC GGG GAT CCG TCG ACC
flhC-FLAG-F	TAT TCC ACA ACT GCT GGA TGA ACA GAT CGA ACA GGC TGT TGGC AGC GGC GAC TAC AAA GAC GAT GAC GAC AAG TAA TGT AGG CTG GAG CTG CTT CG
flhC-FLAG-R	TGA CTT ACC GCT GCT GGA GTG TTT GTC CAC ACC GTT TCG GAT TCC GGG GAT CCG TCG ACC
fliA-FLAG-F	TCA GGC CAT CAA ACG ATT ACG CAC CAA ACT GGG TAA GTT AGGC AGC GGC GAC TAC AAA GAC GAT GAC GAC AAG TAA TGT AGG CTG GAG CTG CTT CG
fliA-FLAG-R	ATA CGT TGT GCG GCA CTT TTC GGG TGC GAT CAT GCG CGA CAT TCC GGG GAT CCG TCG ACC
fliT-FLAG-F	TTC CGG TAT GTT ACT CGT GCC AGA TGC GCC TGG CGC CTC AGGC AGC GGC GAC TAC AAA GAC GAT GAC GAC AAG TAA TGT AGG CTG GAG CTG CTT CG
fliT-FLAG-R	TCT GGA GTA TGG AAG AAT TTT CAT ACG AGA CGG GAA AAT AAT TCC GGG GAT CCG TCG ACC
fliC-FLAG-F	GGC GAA CCA GGT TCC GCA AAA CGT CCT CTC TTT ACT GCG TGGC AGC GGC GAC TAC AAA GAC GAT GAC GAC AAG TAA TGT AGG CTG GAG CTG CTT CG
fliC-FLAG-R	CCT TGA TTG TGT ACC ACG TGT CGG TGA ATC AAT CGC CGG AAT TCC GGG GAT CCG TCG ACC
fliD-comple-F	AAA GGA TCC CCC ACG GTT TCT CAC CGT AA
fliD-comple-R	AAA GCA TGC ATA AGC TTT GAT ACC GCT CG
fliD-L443R-F	GCC CAG TTT ACC CAA CGG GAT ACC ATG ATG AGT
fliD-L443R-R	ACT CAT CAT GGT ATC CCG TTG GGT AAA CTG GGC
fliD-over-F	AAA GGA TCC ATG GCT TCA ATT TCA TCA TT
fliD-over-R	AAA GTC GAC ATA AGC TTT GAT ACC GCT CG
fliT-over-F	AAA GGA TCC ATG ACC TCA ACC GTG GAG TT

fliT-over-R	AAA GTC GAC ATT TTC ATA CGA GAC GGG AA
fliD(1-467)-F	GCT ATA TGG ATC CGG AAA ACC TGT ATT TTC AGG GCA TGG CTT CAA TTT CAT CAT TAG GTG
fliD(1-300)-R	GCT AAT TCT CGA GTC ACT CAA CGG CGG TAT ATT TGG TTA
fliD(1-467)-R	GCT AAT TCT CGA GTC AGG ACT TGT TCA TAG CTG TAA A
fliD(45-467)-F	GCT ATA TGG ATC CGG AAA ACC TGT ATT TTC AGG GCA CCG CCT ATG GCA CAT TGA AAA G
fliD(339-467)-F	GCT ATA TGG ATC CGG AAA ACC TGT ATT TTC AGG GCA AAA CAA TGG CGG AAA TTG GCA TC
fliD(401-467)-F	GCT ATA TGG ATC CGG AAA ACC TGT ATT TTC AGG GCG ACG GCA TTA TTG ATA ATG CGC A
fliD(428-467)-F	GCT ATA TGG ATC CGG AAA ACC TGT ATT TTC AGG GCA GCA TCG ATG AAA CCG TTG CCC
fliT(1-94)-F	GCT ATA TGG ATC CAT GAC CTC AAC CGT GGA GTT TAT
fliT(1-94)-R	GCT AAT TCT CGA GTC ATC CGA TCA AAC TAC TCA GTT CAT C
fliD-Q439R-F	GCC CGT TAC AAG GCC CGG TTT ACC CAA CTG GAT
fliD-Q439R-R	ATC CAG TTG GGT AAA CCG GGC CTT GTA ACG GGC
fliD-L443R-F	GCC CAG TTT ACC CAA CGG GAT ACC ATG ATG AGT
fliD-L443R-R	ACT CAT CAT GGT ATC CCG TTG GGT AAA CTG GGC
pET28a-EGFP-FliD-F	AAA GGA TCC GGC AGC GGC AGC GGC AGC GGC AGC ATG GCT TCA ATT TCA TCA TT
pET28a-EGFP-FliD-R	AAA AAG CTT ATA AGC TTT GAT ACC GCT CG
EGFP-fliD-FLAG-F	AAA GTC GAC ATG GTG AGC AAG GGC GAG GA
EGFP-fliD-FLAG-R	AAA AAG CTT TTA CTT GTC GTC ATC GTC TTT GTA GTC GCC GCT GCC GGA CTT GTT CAT AGC TGT AA
fliD-FLAG-F	AAA GTC GAC ATG GCT TCA ATT TCA TCA TT
fliD-FLAG-R	AAA AAG CTT TTA CTT GTC GTC ATC GTC TTT GTA GTC GCC GCT GCC GGA CTT GTT CAT AGC TGT AA

Table S6. Primers used in qRT-PCR analysis

**Primers**	**Sequences (5' to 3')**
qRT-flhC-F1	ATA TCC AGT TGG CGA TGG AG
qRT-flhC-R1	TTG CTC CCA GGT CAT AAA CC
qRT-flhD-F1	ATC GTC CAG GAC AAA GCA TC
qRT-flhD-R1	TCG TCC ACT TCA TTG AGC AG
qRT-fliA-F1	CCG CTG AAG GTG TAA TGG AT
qRT-fliA-R1	GCT GCA CTG CGT AAG TGG TA
qRT-fliZ-F1	AAC TGC TCG ACC GCA TTA CG
qRT-fliZ-R1	AGT GCA AAT CGC CGC AAA
qRT-fliI-F1	TGC GTC GTT ATG GAC GTT TA
qRT-fliI-R1	CTT CCA GCG GCA TTA GAA AC
qRT-fliJ-F1	CGC TGG ATC AAC TAT CAG CA
qRT-fliJ-R1	GTC GGT CCT GTA AGG TTT GC
qRT-fliM-F1	GGA TAT TAC CGT GGG TGC CAT A
qRT-fliM-R1	GCT TCA GGT GGA TCA GGT TCA
qRT-fliT-F1	ACG GTG ATG GAA AAG CAA AC
qRT-fliT-R1	CTG GCA CGA GTA ACA TAC CG
qRT-fliC-F1	TGA CAG CAG CAG GTG TTA CC
qRT-fliC-R1	CGC CAC CCA GTT TGT TTA GT
qRT-fljB-F1	GCC AAC GAC GGT GAA ACT AT
qRT-fljB-R1	TGC ATC ATC AAG ACC CGA TA
qRT-fliD-F1	CCG TGA CGG TAA CGA AAG AT
qRT-fliD-R1	CCC GGT CTG GAT AGT ACG AA
qRT-gyrB-F1	ATA ACG CCA CGC AGA AAA TGA
qRT-gyrB-R1	TGG CTG ATA CAC CAG CTC TTT G

Reference

1. Lucas, R. L. & Lee, C. A. Unravelling the mysteries of virulence gene regulation in *Salmonella typhimurium*. *Mol. Microbiol.* **36,** 1024-1033 (2000).

2. Woodcock, D. M. *et al.* Quantitative evaluation of *Escherichia coli* host strains for tolerance to cytosine methylation in plasmid and phage recombinants. *Nucleic Acids Res.* **17,** 3469-3478 (1989).

3. Datsenko, K. A. & Wanner, B. L. One-step inactivation of chromosomal genes in *Escherichia coli* K-12 using PCR products. *Proc. Natl. Acad. Sci. USA* **97,** 6640-6645 (2000).

4. Chang, A. C. & Cohen, S. N. Construction and characterization of amplifiable multicopy DNA cloning vehicles derived from the P15A cryptic miniplasmid. *J. Bacteriol.* **134,** 1141-1156 (1978).

5. Soncini, F. C., Véscovi, E. G. & Groisman, E. A. Transcriptional autoregulation of the *Salmonella typhimurium phoPQ* operon. *J. Bacteriol.* **177,** 4364-4371 (1995).

6. Kong, M. *et al*. A novel and highly specific phage endolysin cell wall binding domain for detection of *Bacillus cereus*. *Eur. Biophys. J.* **44,** 437-446 (2015).
